# Single-Cell RNA Sequencing in Organ and Cell Transplantation

**DOI:** 10.3390/bios14040189

**Published:** 2024-04-11

**Authors:** Roozbeh Abedini-Nassab, Fatemeh Taheri, Ali Emamgholizadeh, Hossein Naderi-Manesh

**Affiliations:** 1 Faculty of Mechanical Engineering, Tarbiat Modares University, Tehran P.O. Box 1411944961, Iran; 2 Biomedical Engineering Department, University of Neyshabur, Neyshabur P.O. Box 9319774446, Iran; 3 Department of Nanobiotechnology, Faculty of Bioscience, Tarbiat Modares University, Tehran P.O. Box 1411944961, Iran; naderman@modares.ac.ir; 4 Department of Biophysics, Faculty of Bioscience, Tarbiat Modares University, Tehran P.O. Box 1411944961, Iran

**Keywords:** single-cell RNA sequencing, transplantation, microfluidics, biomarkers

## Abstract

Single-cell RNA sequencing is a high-throughput novel method that provides transcriptional profiling of individual cells within biological samples. This method typically uses microfluidics systems to uncover the complex intercellular communication networks and biological pathways buried within highly heterogeneous cell populations in tissues. One important application of this technology sits in the fields of organ and stem cell transplantation, where complications such as graft rejection and other post-transplantation life-threatening issues may occur. In this review, we first focus on research in which single-cell RNA sequencing is used to study the transcriptional profile of transplanted tissues. This technology enables the analysis of the donor and recipient cells and identifies cell types and states associated with transplant complications and pathologies. We also review the use of single-cell RNA sequencing in stem cell implantation. This method enables studying the heterogeneity of normal and pathological stem cells and the heterogeneity in cell populations. With their remarkably rapid pace, the single-cell RNA sequencing methodologies will potentially result in breakthroughs in clinical transplantation in the coming years.

## 1. Introduction

Single-cell ribonucleic acid sequencing (scRNA-seq) is now considered the state-of-the-art technology for assessing the function of individual cells within a large tissue sample. It is a powerful technique for profiling the expression of genes in individual cells, providing a new way to study biological processes and identify rare cell types. This analytical method has found application across various domains, including cancer biology [1,2], neurobiology [3,4], immunology [5,6], and transplantation [7,8]. In areas such as cancer research, scRNA-seq allows scientists to understand how tumors differ at the cellular level and how they develop over time. Similarly, in neuroscience, scRNA-seq can be used to uncover how different neural circuits are formed and regulated, providing new insight into the development and functioning of the brain. In addition, scRNA-seq can be used to study the diversity of microbial populations within complex environments, such as the human gut [9]. Recently, scRNA-seq has also been used to study the role of graft–host interaction after transplantation and for preclinical evaluation of the grafts and therapeutic stem cells to ensure reliable and safe transplantation [10].

The development of scRNA-seq is partly due to the recent advances in the field of biomicrofluidics. In particular, droplet-based microfluidic chips, in addition to various applications in different fields [11], offer the encapsulation of thousands of single-cells and barcode-carrying beads in droplets, where the genomic data of the single cells can be captured [12]. After being captured with barcoded beads The messenger ribonucleic acids (mRNAs) of individual cells are sequenced. The obtained data are then analyzed to find the gene expression level at the single-cell resolution.

Currently, transplantation remains the primary solution for organ failure; however, associated biological challenges still hinder its success rate [13]. Monitoring the graft health and interaction with the host to detect post-transplant complications and possibly prevent them is an essential need in determining the long-term success of the transplantation and a key component of transplant therapy. While solid biopsy is the gold standard for allograft monitoring, analyzing body fluids, such as blood and urine, is also becoming popular as a less invasive and more patient-friendly method capable of reliably detecting transplant complications. As such, scRNA-seq is being used to evaluate post-transplant graft health by analyzing the gene expression profile and its changes. By applying scRNA-seq on allograft biopsies at various time points, the transcriptomic profiles of cells in rejection, as well as the changes in cell interactions after anti-rejection treatments, can be identified [14]. This type of analysis can be used to assess the degree to which the graft is compatible with the host’s tissue and can help to identify potential problems that could lead to graft rejection.

In addition to providing insights into the transplantation quality and the graft–host interactions, scRNA-seq enables the discovery of new biomarkers to predict transplantation outcomes. These biomarkers can then be used to assess the likelihood of a successful outcome prior to the transplantation procedure.

Stem cell transplantation is considered another area where scRNA-seq could have a transformative impact. Stem cells regularly divide to maintain, develop, and repair the organs and tissues [15]. In stem cell transplantation, stem cells from a donor are transplanted into a recipient in order to treat a wide range of diseases and injuries, such as leukemia and spinal cord injuries. Unfortunately, the transplantation process is not always successful. Numerous factors can affect the success of the transplantation, such as the quality of the stem cells, the compatibility of the donor and recipient, and environmental factors (e.g., diet and pollutants).

By applying scRNA-seq to stem cell transplantation, it is possible to gain a better understanding of stem cell biology and investigate how the various factors can influence their behavior. This approach would enable researchers to develop and optimize more efficient and successful transplantation protocols and to improve stem cell selection methods. It also could open the door to a new era of personalized, targeted therapies that are more effective and efficient than ever before.

Another application of scRNA-seq is evaluating transplanted tumors for research purposes. The transplantation of tumor tissue into animal models is an established field for studying the effect of the surrounding microenvironment on tumor behavior, including tumorigenesis and metastasis [16]. The scRNA-seq technology captures this information from the transplanted tumor at single-cell resolution.

In this review, we first discuss advanced available scRNA-seq methods and compare them. Then, we delve into the application of scRNA-seq in solid transplantation, including heart, kidney, lung, and liver. Next, we review the use of scRNA-seq in stem cell transplantation. Then, we discuss selected studies where scRNA-seq addresses questions in tumor transplantation. In each section, essential information and the relevant comparisons are tabulated.

## 2. Single-Cell RNA Sequencing

As an emerging technology used to measure expression levels of genes within individual cells, scRNA-seq offers an unparalleled level of resolution, allowing scientists to gain a deeper understanding of complex biological processes, such as cellular heterogeneity and cellular interactions. The scRNA-seq approaches are generally classified into two broad categories: plate-based and microfluidic-based methods. Microfluidic tools offer novel sequencing methods with significant advantages, including fast handling, labor saving, and high throughput. Thus, these technologies have been widely used recently. The basic principle of scRNA-seq is to capture and sequence the mRNA molecules present within individual cells. By doing so, it is possible to quantify the expression levels of various genes, providing a snapshot of the transcriptome of each cell. The acquired sequencing data can then be used to infer biological characteristics such as the cell type and its state of activation. In addition, it offers insights into the dynamics and interactions of molecular networks within individual cells. 

To obtain the mentioned data from the single cells, single-cell transcriptome analysis typically involves three main steps [17]. The first step is to dissociate and isolate live single cells from tissues and keep them alive. They need to be protected against gene and protein expression variations due to cell dissociation and handling. This step may be performed using mechanical and enzymatic-based methods. The obtained single cells may then be selected based on their membrane protein expressions using fluorescence-activated cell sorting. The second step is to reverse-transcribe mRNA selectively to achieve a cDNA library. This step involves sub-steps of RNA capture, reverse transcription, RNA sequencing, and library construction. The third step is to sequence and analyze the cDNA library obtained from the single cells. Data analysis involves several sub-steps, including quality control, preprocessing, quantification, dimensionality reduction, and visualization. Early errors in data not removed during the preprocessing steps and quality checks may propagate throughout the rest of the analysis. Dead cells with damaged cell membranes, cell doublets, or bead doublets need to be detected and filtered. For example, the leakage of mRNAs from the damaged membranes has a substantial impact on the overall read counts. Also, cDNA amplification may introduce an unwanted bias toward the amplification of cDNA transcripts with specific sizes. 

### 2.1. Single-Cell Isolation Methods

In order to isolate single cells for lysis and analysis, different methods are used. Here, we mention six widely used cell isolation methods, four of which are based on microfluidic tools.

#### 2.1.1. FACS

Fluorescence-activated cell sorting (FACS) systems sort individual particles and cells based on fluorescent signals obtained from them [18,19]. It is possible to use these systems in single-cell analysis methods to isolate fluorescently labeled single cells based on their specific surface molecules. The fluorescent labels are excited with the means of a laser beam, after which the cells are sorted into specific microwells of interest [20] for further steps toward scRNA-seq. The advantages of cell sorting based on FACS include their high accuracy. However, they are not typically suitable for sorting samples with low cell numbers.

#### 2.1.2. Micromanipulation

Mechanical micromanipulation is considered a traditional method to put individual particles into separate chambers to be studied. This technique is based on manual cell micropipetting under a microscope or based on pipetting robots. In these methods, the cell concentration in the cell suspension is lowered to ensure pickup at single-cell resolution. Mechanical micromanipulation is widely used in biological research; however, it needs skilled operators, it is low-throughput, and the cells may be damaged [21,22,23].

#### 2.1.3. Passive Hydrodynamic Trap-Based Microfluidics

Microfluidic chips provide good control of small volumes of fluids, as well as tiny objects. Hence, cell handling based on these devices is developed as a more modern technique. Hydrodynamic trap-based microfluidic chips are one of these methods that are widely used in the field [24,25,26]. In this method, the cell suspension is injected into the microfluidic device, where the single cells are captured in the trap sites. By entering a cell into a trap site, the hydrodynamic resistivity of that path increases, limiting the occupation of that trap with the other cells. This passive method based on hydrodynamic forces does not require additional forces to manipulate the particles; however, the unwanted shear stress applied to trapped cells may alter the cell behavior [27]. Also, the cell concentration needs to be controlled to avoid channel clogging. Since the traps are not typically isolated, another challenge is the high chance of cell content contamination. Recently, a method for trapping cell-encapsulating droplets has been introduced, answering most of the mentioned drawbacks of the old systems [28]. In this system, the cells do not experience high shear stresses, and the droplet acts as an isolated microchamber, preventing unwanted cross-contaminations.

#### 2.1.4. Active Cell Manipulation Microfluidics

Microfluidic chips enhanced with active particle manipulation methods use at least one force other than hydrodynamic forces to manipulate cells. Methods based on magnetic [29,30,31,32,33,34], electric [35,36,37,38], acoustic [39,40,41,42,43], and optical forces [44,45,46,47,48], each of which, with their advantages and disadvantages, have been developed. In general, the active methods are rather more complicated than the passive methods; however, they typically offer more precise control of individual cells. Magnetic-based techniques require cell magnetic nanoparticle labeling, while other methods do not. There are cell mutation concerns in optical manipulations [49]. Also, they may have difficulties in opaque environments. The methods based on electric forces (e.g., electrowetting [50,51]) require complex wiring systems. In acoustic systems, manipulating specific single particles is typically not offered. Microfluidics chips employing a combination of different techniques have shown promising results [52,53]. These methods borrow desired specifications from different techniques to offer unique capabilities, while undesired aspects are limited. For example, a recently developed device called the magnetomicrofluidic chip assembles single cells in semi-isolated low-shear microchambers in a microfluidic chip using the combination of hydrodynamic traps and magnetic or electric forces [29,54,55]. In these systems, the undesired shear stress and the cell content contamination seen in hydrodynamic trapping systems are eliminated, while they provide high-throughput precise particle manipulation. 

#### 2.1.5. Valve-Based Microfluidics

To control the fluid flow inside the microchannels in a microfluidic chip, microvalves are used [56]. Typically, the microvalves have been developed using a multilayer microfluidic structure [57]. Quake valves that control the flow rate in a fluid-carrying channel by applying a pneumatic pressure on a PDMS membrane on top of that channel to deflect it are considered one of the most common microvalve designs [57]. Based on these valves, hundreds of individually addressable microchambers on a single chip are fabricated [58], where single cells can then be stored for scRNA-seq [59]. The microchambers can be fully isolated using these chambers; however, if the cells are punched under a pressed valve, their contents will be dispersed throughout the microchannels.

#### 2.1.6. Droplet-Based Microfluidics

Among various microfluidic single-cell isolation techniques, droplet-based microfluidics have drawn significant attention with applications in scRNA-seq. In this method, individual cells are encapsulated into droplets acting as tiny microchambers [60,61,62,63]. Various microfluidic chip designs for producing microdroplets have been proposed. These designs are generally categorized into three groups: co-flow [64,65], cross-flow [66,67,68], and flow focusing [69,70]. 

### 2.2. scRNA-Seq Methods

**Figure 1:**
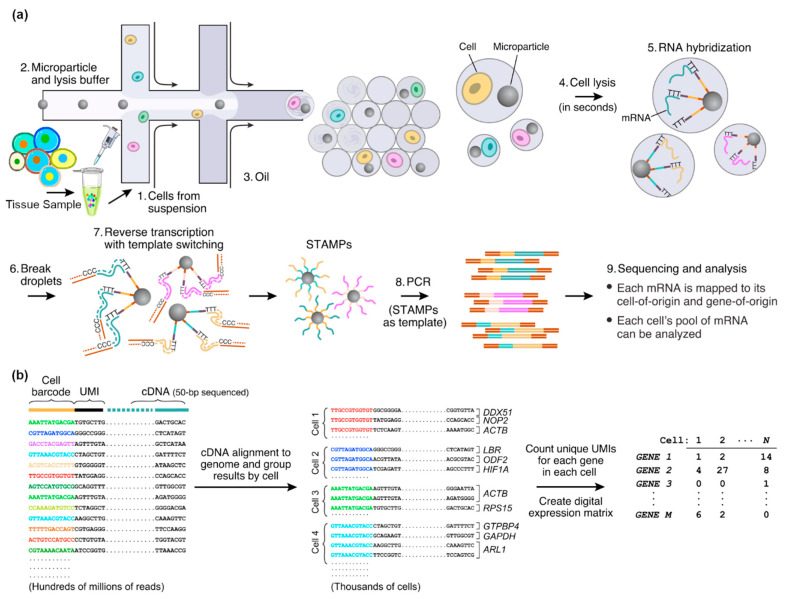
Schematic of the Drop-seq single-cell RNA-sequencing steps is illustrated. (**a**) The library preparation steps are shown. Single cells are separated from a tissue to form the single-cell suspension, which then forms the first aqueous phase to be injected into the microfluidic chip. The other aqueous phase carries the barcoded beads suspended in a lysis buffer. The droplet-based microfluidic chip in a T-junction joins these two aqueous flows, where they form the discrete phase (i.e., droplets encapsulating the required materials) in an oil continuous phase. In the formed microdroplets, the single cells are lysed, and their mRNAs are captured with the primers on the microparticles. Then, the droplets are broken, and the collected mRNAs are reverse-transcribed, forming STAMPs. After PCR, NGS and analysis are performed. (**b**) The analysis steps after sequencing are shown. Sequencing reads are aligned to a reference genome to find the gene of origin of the cDNA. Also, they are organized based on their cell barcodes to count UMIs for each gene in each cell, based on which the expression matrix is extracted. Reprinted from [60], with permission from Elsevier Copyright 2015 Elsevier Inc.

Drop-seq is one of the widely used scRNA-seq methods that use droplet-based microfluidic chips. It was developed by Dr. Macosko and was first introduced in 2015 [60]. Figure 1 illustrates a schematic of typical steps in Drop-seq. A flow-focusing droplet-based microfluidic chip is used to encapsulate single cells with single barcode-carrying beads into microdroplets. The microdroplets are formed at a cross-junction, where cell and bead suspension (to form the dispersed phase) and the oil (the continuous phase) meet. The dispersed passes through the junction and expands into the main channel. At the cross-junction, the continuous phase forms a neck in the dispersed phase, which then shrinks until it breaks up and the droplet forms. Based on the ratio of the viscous force to the inertial tension force, various droplet generation regimes may be observed (e.g., squeezing, dripping, or jetting). In Drop-seq and other droplet-based single-cell analysis tools, the dripping regime is commonly used to form highly monodispersed droplets. 

After droplet formation, the lysis buffer breaks the cell membrane, and their mRNAs, which are then captured with the barcode-carrying beads, are released. These single beads in each droplet contain oligonucleotides and unique molecular identifiers (UMIs). They contain primers for the reverse transcription of the mRNAs captured from single cells, and the resulting cDNA is sequenced to uncover the gene expression profile. The resulting sequencing data are then used to study the expression of thousands of genes within cells at the single-cell level. Compared with the old protocols, Drop-seq offers numerous advantages, including high throughput and reasonable cost per cell [71]. These features allow researchers to analyze tens of thousands of single cells in an experiment. This capability can be utilized to study cell heterogeneity, signaling pathways, and developmental trajectories. Drop-seq has been widely used in the biology scientific community and has fundamentally contributed to our understanding of single-cell biology and various diseases.

The data obtained from the sequencing need to be computationally analyzed. Because of the complexity of the information generated by scRNA-seq, a dimensional reduction step is needed before visualizing it. Some popular dimensional reduction methods are principal component analysis (PCA), t-Stochastic Neighbor Embedding (t-SNE), and Uniform Manifold Approximation and Projection (UMAP). The goal of these methods is to keep the most influential features and omit the ones with less impact. After recusing the dimension of the data, a unique coordinate is typically assigned to each cell such that the ones with similar genetic information are placed in close proximity to be clustered based on methods such as the k-nearest neighbor [17].

In Drop-seq, in order to prevent more than a single bead from being in a droplet, its concentration is lowered. However, numerous droplets then lack beads, which may not be a major problem in normal analysis. However, when the cell numbers in samples are limited, losing cells in droplets with no bead becomes challenging. To overcome this problem, InDrop technology is proposed [72]. This method is similar to Drop-seq, but it uses barcoded hydrogels as a more complex barcoding system compared with the beads, which allows for higher throughput and better cell type identification. In this method, high concentrations of hydrogels can be injected into the microfluidic chip, while their size and flexibility prevent having more than a single hydrogel in a droplet.

The gel beads in emulsion (GEMs) utilized in InDrop find analogous applications in the 10× Genomics Chromium system, which is another well-known tool used for scRNA-seq. Similar to Drop-seq, this method uses a microfluidic system to encapsulate individual cells with barcode-carrying beads and perform reverse transcription and cDNA amplification. The system is high-throughput and relatively easy to use, with a straightforward sample preparation protocol and data analysis workflows. Moreover, the system is compatible with a variety of sample types, including both fresh and frozen tissues, as well as cultured cells. The 10× Genomics Chromium system has been used in a variety of applications, including the identification of rare cell types, the characterization of complex tissues and organs, and the study of developmental processes and disease progression [12,73]. Drop-seq, InDrop, and 10× Genomics are widely considered the most successful scRNA-seq technologies in the field. 

Seq-well is another scRNA-seq technology that was introduced in 2017 [74]. The Seq-well technology uses a microfluidic chip with thousands of individual wells. Each well is designed to capture a single cell and a single bead, where the cell is then lysed, and its RNA is captured with the bead and barcoded using UMIs. Similar to the methods mentioned above, the resulting cDNA is then amplified and sequenced using next-generation sequencing technology. Since this method does not require any specialized equipment, it is accessible to many researchers. However, because the microfluidic chip is loaded with cells and beads using pipets, no control over individual particles has been offered. As a result, the microwells may be filled with more than one cell or bead, which may be challenging.

In some scRNA-seq methods, as opposed to the microfluidic systems, microplates are used. Smart-seq is one such example that captures the full-length RNAs of single cells and amplifies them for high-throughput sequencing [75,76]. In this method, the cells are lysed in a hypotonic solution, and RNAs are converted to full-length cDNAs. After PCR amplification, the full-length cDNAs are used to construct standard Illumina sequencing libraries. Its updated version, called Smart-seq2, was developed in the lab of Dr. Sandberg and was first published in 2014 [77,78]. In Smart-seq2, reverse transcription (RT), template switching, and preamplification were optimized to offer an increased cDNA yield from single cells, better sensitivity, and enhanced repeatability. 

Smart-seq3 combines full-length transcriptome coverage with a 5′ unique molecular identifier RNA counting strategy that enables the in silico reconstruction of thousands of RNA molecules per cell [79]. This technique is suitable for analyzing samples with limited cell numbers. In 2020, Smart-seq3, which combines full-length transcriptome coverage with a 5′ unique molecular identifier RNA counting method, came out. With Smart-seq3, more transcripts per cell compared with Smart-seq2 can be captured. All Smart-seq methods can be enhanced by providing more precise control over individual cells using microfluidic systems such as fLuidigm C1.

Another plate-based scRNA-seq method, in which FACS sorts single cells into 96- or 384-well plates, is the single-cell combinatorial indexed RNA sequencing (sci-RNA-seq) technique. It uses combinatorial cell barcoding and UMIs to enable the high-throughput analysis of large numbers of cells with high sensitivity and accuracy [80]. By barcoding individual cells with a unique combination of DNA tags, the method allows for the identification and quantification of each transcript in each cell. One key advantage of sci-RNA-seq is its ability to offer full-length transcript coverage. This feature allows the identification of novel splice variants and the accurate quantification of transcript isoforms. The method also offers high sensitivity and the detection of low-abundance transcripts and rare cell types.

MATQ-seq (Microbial RNA-Seq for Meta-Transcriptomics and -Transcriptome Analysis) is another high-throughput sequencing-based method, which is mostly used in a mixed microbial community [81]. This technique is based on pipetting single cells into PCR tubes and uses selective hybridization methods to capture mRNA, followed by cDNA synthesis, amplification, and sequencing steps. The PCR amplification step is replaced by in vitro transcription (IVT) in a method called CEL-Seq to increase efficiency [82]. Then, Cell-seq2, which has been optimized for higher sensitivity and less hands-on time, was introduced [83]. Although Cell-seq uses single-cell pipetting, Cell-seq2 works based on either robotic liquid handles or a Fluidigm C1 microfluidic chip. 

Nanopore sequencing is one of the most promising techniques that offers long reads. In this approach, while the biomolecule zips through a nanopore in a membrane, it changes a characteristic identifying the biomolecule sequences [84]. The MinION (Oxford Nanopore Technologies, Oxford, UK) is one of the earliest commercialized real-time, portable nanopore sequencers to have been widely used. Single-cell RNA sequencing based on nanopore devices has also been presented [85,86]. Thus, this tool has the potential to be used in studying transcriptomes in transplanted organs.

Each of the mentioned methods has its own advantages and disadvantages, and the choice of method depends on the application of interest, the biology research question, and the available resources. Scientists may choose a method based on parameters such as cost, throughput, sensitivity, full-length transcript coverage, and compatibility with their samples. Table 1 lists the advantages and disadvantages of some of the important scRNA-seq methods.

## 3. Single-Cell RNA Sequencing in Organ Transplantation

In this section, various organ transplantations in which scRNA-seq is used are discussed.

### 3.1. Heart Transplantation

**Figure 2:**
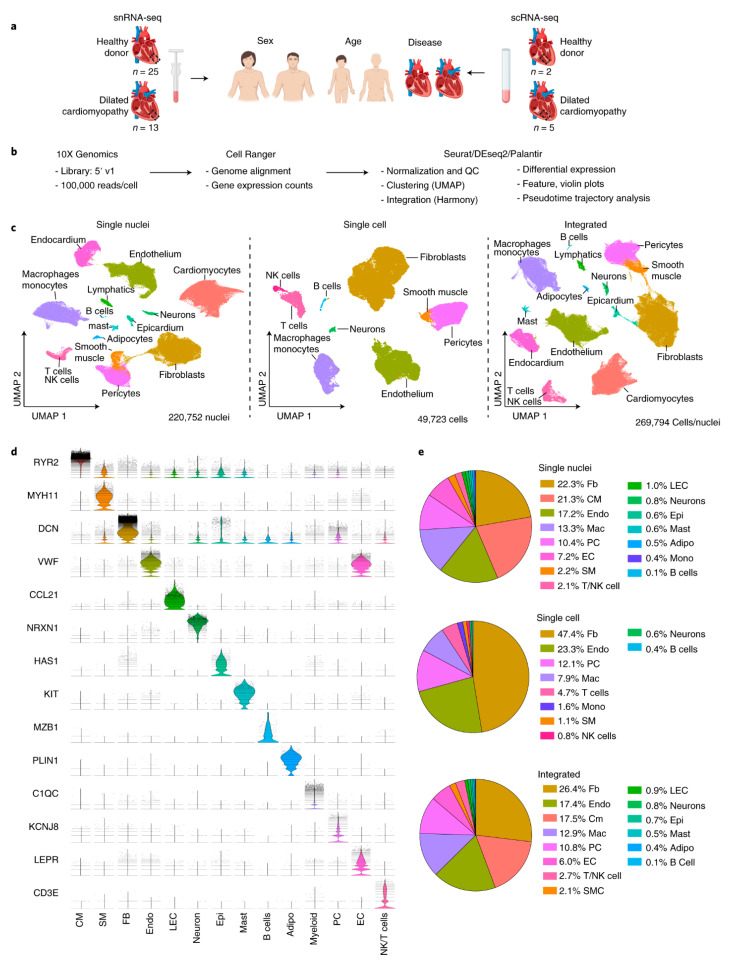
(**a**) Schematic representing the single-nucleus RNA-sequencing and single-cell RNA-sequencing experiments of heart tissues. (**b**) The analysis pipeline is depicted. It contains tissue preprocessing, library generation, alignment (Cell Ranger), and further data analysis. (**c**) Clustering. (**d**) Violin plots based on analyzing the integrated dataset showing characteristic biomarkers of identified cell populations. (**e**) Pie chart presenting the cell proportion. The figure is taken from [91] with permission under a Creative Commons Attribution 4.0 International License (http://creativecommons.org/licenses/by/4.0/, accessed on 1 February 2024).

By identifying the cellular composition of the cardiac system and the cell states before and after transplantation, crucial information about the graft can be obtained. This idea is investigated by using the scRNA-seq technique to find the single-cell transcriptomic atlas of human cardiac arteries and identify the cellular compositions in various cardiac arteries [91]. In these experiments, various cell populations, including vascular smooth muscle cells, fibroblasts, myofibroblasts, macrophages, T cells, B cells, endothelial cells, natural killer cells, mast cells, and oligodendrocytes are detected. These achievements are useful as a reference to find disease-associated cell populations in vascular and heart diseases. As shown in Figure 2, they have combined scRNA-seq (*n* = 7) with single-nucleus RNA-sequencing (snRNA-seq) (*n* = 38) to obtain data from heart samples (left ventricular (LV) tissues from 27 healthy donors and 18 patients with dilated (nonischemic) cardiomyopathy). 

In a similar study, researchers used scRNA-seq technology to identify intra-graft cell heterogeneity in acute heart allograft rejection in mice [7]. They employed the 10× Chromium platform, based on which about 2000 genes in almost 20,000 cells from two allogeneic heart grafts and about 20,000 cells from two syngeneic heart grafts were detected. They used this technique to identify the cell types (i.e., 21 distinct cell populations) and states associated with acute rejection and introduced the potential predictive biomarkers. They reported five cell clusters, including two resident macrophage groups, two infiltrating macrophage groups (predominantly from allogeneic grafts, one in an active state and one silent), and one dendritic cell-like monocyte group. The authors re-clustered endothelial cells into five subclusters, one of which was from allogeneic grafts. This cell population showed expression of Ubiquitin D, which they claimed was upregulated in heart and kidney rejection. Endothelial cells have been found by other researchers to show tissue-specific identities and unique transcriptomic signatures as well [92].

In another work, researchers used scRNA-seq to study transplant arteriosclerosis as a key challenge in long-term transplantation survival [93]. They used unbiased clustering analyses on a mouse model to identify 21 cell clusters at various disease stages and some novel subpopulations enriched in the allografts. They also reported the local formation of tertiary lymphoid tissues and possible intra-graft alloimmune response modulation. They reported the potential role of Ccl21a and Cxcr3 in regulating early chemotaxis and immune cell infiltration. They also used single-cell analysis to compare the immune response in mouse allograft and the atherosclerosis models. Researchers claim that their results depict both similarities and differences in atherosclerosis models and allograft-induced transplant arteriosclerosis. The innate and adaptive immune responses exist in the two models; however, distinct cell subpopulations may mediate the responses. 

Based on the scRNA-seq results in a preprint [94], donor and recipient macrophage populations co-exist within the heart allograft. They claim that donor CCR2+ macrophages play a key role in allograft rejection, and MYD88 signaling inhibition in donor macrophages suppresses the allograft rejection. These results suggest that the signals from the transplanted graft from the donor and not the signals from the recipient macrophage populations define the destiny of the patient.

Researchers found cell-specific transcriptional signatures that are associated with age and heart failure [91]. They realized that cardiomyocytes go to common disease-associated cell states; however, fibroblasts and myeloid cells become diverse. They also found that endothelial cells and pericytes show transcriptional shifts.

Researchers compared cellular composition in various arteries in heart-transplanted patients and realized that some artery-specific vascular smooth muscle cell and fibroblast subpopulations exist [95]. In healthy conditions, the communication between vascular smooth muscle cells and fibroblast is reported to be dominant. They reported the enrichment of atherosclerosis-associated genes in endothelial cells and macrophages. They also reported that intercellular communication between endothelial cells and immune cells may increase during atherosclerosis. Based on this study, they believe that interactions between ICAM1/VCAM1 (EC1) and ITGB2 (immune cells, especially inflammatory macrophages) are important factors in the pathogenesis of atherosclerosis.

Various studies have shown the power of scRNA-seq in identifying previously unknown cell types and gene expression profiles in heart transplantation. In these experiments, different donor and receptor species have been considered. In some studies, human tissues are transplanted, and in other animal models of heart transplantation, they are demonstrated. In Table 2, some examples of these studies with their key findings are tabulated. 

### 3.2. Kidney Transplantation

The scRNA-seq technique has fundamentally leveraged our knowledge of renal cell identities and their genomic biomarkers [106]. These achievements identify the pathophysiology of kidney conditions, including early diabetic nephropathy [107], tumor compositions [108], and allograft rejection [5].

Multiple cell populations and subpopulations that are available in the kidney and scRNA-seq can identify this cellular heterogeneity at single-cell resolution. This identification can be enhanced by the integration of single-cell transcriptome and chromatin accessibility datasets. snRNA-seq and single nucleus assay for transposase-accessible chromatin using sequencing are used to generate cell-type-specific chromatin accessibility and transcriptional profiles of the kidney. Researchers have shown that most of the accessible chromatin regions are closely associated with the expressed genes. This multi-omics method allows the detection of unique cell states within the cellular population in the kidney [109].

**Figure 3:**
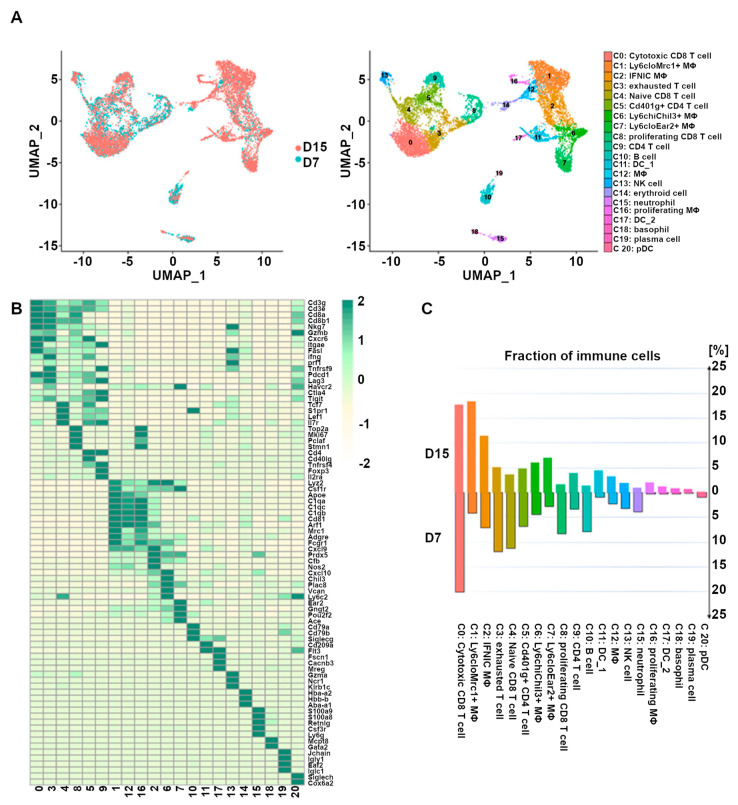
The presence of 20 CD45+ immune cell clusters is detected based on the scRNA-seq of mice kidney allograft. (**A**) UMAP plots of cell clusters identified based on biomarkers. Each dot represents a single cell, with its color demonstrating the cluster designation. (**B**) Heatmap representing the biomarkers of each cluster of kidney graft immune cells. (**C**) Bar plot demonstrating the proportions of the 20 identified immune cell populations in the kidney allografts obtained 7 days and 15 days after transplantation, respectively. The colors are chosen according to clusters in (**A**). UMAP, Mφ, DC, NK, and pDC stand for Uniform Manifold Approximation and Projection, macrophage, dendritic cell, natural killer, and plasmacytoid dendritic cell, respectively. The figure is taken and reproduced with permission from [110] under the terms of the Creative Commons Attribution License (CC BY) (http://creativecommons.org/licenses/by/4.0/, accessed on 1 February 2024).

In a recent study, a mouse kidney allograft rejection model and scRNA-seq were used to study CD45+ leukocytes in allografts on days seven and fifteen after transplantation [110]. Researchers detected 20 immune cell types (See Figure 3) and found that macrophages and CD8+ T cells made the main cell populations at both time points. They reported that in the transition from acute rejection toward chronic rejection, the proportion of CD8+ T cells dropped. However, the proportion of macrophages and dendritic cells highly increased, with Ly6c^lo^Mrc1^+^ and Ly6c^lo^Ear2^+^ macrophages being the main subgroups. They concluded that the drop in CD8+ T cells, B cells, and neutrophils and the rise in Ly6c^lo^Ear2^+^ and Ly6c^lo^Mrc1^+^ macrophages contribute to the transition from acute rejection to chronic rejection. Clonal CD8+ T cell responses have been reported to show important roles in rejection [111].

Another research group employed kidney transplant biopsies and combined germline DNA sequencing with scRNA-seq to analyze the transcriptional profiles of donor- and recipient-derived leukocytes in acute antibody-mediated rejection and non-rejecting states [112]. They identified the major kidney cell types, as well as lymphocytes and macrophages. The ratio of leukocyte donor/recipient can be utilized as a rejection status indicator. They found that macrophages and T cells have distinct transcriptional profiles between the donor and recipient groups. They also claim that donor macrophages persist for years after graft transplantation.

Another group used scRNA-seq to identify 16 cell types and states in a human kidney biopsy specimen [5]. By comparing the results from a healthy adult kidney and a kidney transplant biopsy, they identified rejection-related, segment-specific proinflammatory responses. Of the three endothelial cell subclusters they found, two groups were active, one of which expressed the Fc receptor pathway and Ig internalization genes, which stands for antibody-mediated rejection.

Similarly, scRNA-seq has been applied to three healthy human kidneys and two chronic kidney transplant rejection (CKTR) biopsies [113]. Based on unsupervised clustering analysis of the obtained data, they identified 15 cell types (e.g., five natural killer T cell subclasses, CD4+ T cells, CD8+ T cells, cytotoxic T lymphocytes, regulatory T cells, natural killer cells, two memory B cell subtypes, CD14+ and CD16+ monocyte groups, and a novel subpopulation (myofibroblasts) in fibroblasts expressing collagen and extracellular matrix components). They also distinguished the CKTR group by the higher numbers of immune cells and myofibroblasts. The identification of the functional differences between the cell subpopulations and between samples from healthy and graft-rejected patients was based on the single-sample gene set enrichment (ssGSEA) algorithm. Other groups found a B cell subset (CD19^+^IGLC_3_^low^IGKC^high^TCL_1_A-CD_127_^+^) to be much lower in renal allograft recipients with accommodation (i.e., allograft recipients that are neither rejected nor infected after immunosuppression) than that in healthy people [114]. 

B cell subsets have attracted the attention of other researchers as well. Clark and coworkers showed that in kidney rejection, infiltrating B cells contributes to specific innate signaling pathways, which may be related to inflammation [115]. In particular, they showed that B-innate cells generate inflammation-specific antibodies and drive local inflammation in transplanted kidney rejection.

Researchers believe that myeloid cells play a key role in transplant rejection [116,117]. They used scRNA-seq to study the murine kidney transplantation model to study the contribution of these cell subsets and their signaling pathways in graft rejection [118]. They showed that kidney allograft-infiltrating myeloid cells differentiate from monocytes to proinflammatory macrophages. They also identified Axl as a key gene in the differentiation of proinflammatory macrophages in transplanted kidneys, which also promotes the differentiation and proliferation of donor-specific T cells. scRNA-seq analysis has also been used to detect and study the type and status of monocytes/macrophages in kidney transplantation [119]. They form two different subpopulations: resident and infiltrating monocytes/macrophages. The number of resident macrophages decreases during kidney rejection. In these macrophages, the relevant genes during phagocytosis are upregulated.

The scRNA-seq technique has also been used in kidney organoid transplantation [120]. Human-induced pluripotent stem cell (iPSC)-derived kidney organoids have multiple nephron-like structures that show some renal functions [121]. Although these organoids have attracted much attention in disease modeling and drug screening applications, their reproducibility and reduction in off-target cell generation is a challenge. Researchers have used scRNA-seq to answer this need [120] and showed that cell proportion variations exist between different iPSC lines mainly because of off-target cells. They also analyzed transplanted organoids in mice and realized that off-target cells diminish after transplantation.

In addition to the transcriptomic profile of the cell types within the allograft, spatial transcriptomics in acute kidney injury is important and affects the cells heterogeneously. Researchers identified the spatial transcriptomic signature of ischemia/reperfusion injury and cecal ligation puncture as two murine acute kidney injury models [122]. They realized that spatial reduced expression is associated with the injury pathways. They used scRNA-seq to find epithelial cell–immune dialogue in kidney injury spatially. 

Single-cell RNA sequencing has also been applied to kidney solid biopsies and CD14^+^ peripheral blood mononuclear cells (PBMCs) obtained from Immunoglobulin A nephropathy (IgAN), and the results have been compared to sequences of normal samples [123]. This analysis is used to study the molecular events in IgAN progression that can be used in disease treatment. In IgAN, JCHAIN upregulation is related to the in situ dimerization and deposition of IgA1. The pathological mesangium also indicates high cell–cell communication between renal parenchymal cells and immune cells. Also, the expression of genes specific to kidney-resident macrophages and CD8+ T cells depict abnormal regulation related to proliferation and inflammation. 

Although analyzing kidney biopsy samples has resulted in valuable information, biopsy-associated complications, biopsy specimen scoring variability, invasiveness, and costs are considered concerns in the field. Also, studying the kidney behavior at multiple time points after transplantation to understand its dynamic behavior is an important need; however, repeated biopsies are not convenient and safe, if practically possible [124]. Urinary and PBMCs from kidney transplant recipients are other sources of organ injury biomarkers [125]. Transcriptomic analysis and clustering of these cells are considered noninvasive analysis methods on which researchers are working. Muthukumar, from Weill Cornell Medical College, and his coworkers performed scRNA-seq on urinary cells obtained from kidney transplant recipients, with biopsies classified as acute T cell-mediated rejection, chronic active antibody-mediated rejection, and normal conditions [126]. They claimed that urine samples matched to the acute T cell-mediated rejection biopsy showed increased macrophages, dendritic cells, T cells, and NK cells, while the one matched to normal biopsy displayed dominant kidney tubular epithelial cells. This approach is an innovative method for uncovering the complex cellular landscape of kidney allograft rejection at the single-cell resolution.

Researchers have generated the profiles of various PBMC cell types and their gene expression using scRNA-seq in chronic antibody-mediated rejection patients [127]. Based on their results, MT-ND6, CCL4L2, CXCR4, NFKBIZ, DUSP1, and ZFP36 were upregulated in these patients. They also reported that MAPK and NFκB signaling pathways were activated. They claimed that single-cell sequencing is a potential strategy for understanding the details of the peripheral blood lymphocyte in chronic antibody-mediated rejection patients. 

It has been shown that the integration of spatial and single-cell transcriptomics can localize the cell–cell communication between the epithelial and immune cells [128] in kidney injury. This method is used to spatially map the transcriptomic signature of acute kidney injury in murine models, which can also be applied to human kidney tissue [122]. In Table 3, some key findings of applying scRNA-seq on kidney transplantation are listed. Also, some genes play the biomarker role for transplant problems. In Table 4, some biomarkers in different kidney transplantation studies are tabulated.

### 3.3. Lung Transplantation

Identifying the donor and recipient cells in transplant biology is an important task that is possible with scRNA-seq. One algorithm toward this goal is called scTx, which identifies the donor and recipient genotypes using expressed single nucleotide variants and assigns the cells to a genotype [135]. The authors tested their proposed algorithm on lung transplanted samples and claimed that it could detect two genotypes from post-transplant bronchoalveolar lavage and lungs with chronic lung allograft dysfunction samples. 

Because of chronic rejection, bronchiolitis obliterans syndrome is a big challenge and the key reason for weak lung transplantation outcomes. In a recent work [136], researchers used single-cell RNA sequencing to provide an atlas of bronchiolitis obliterans syndrome after lung transplantation outcomes. This atlas can be used to identify the changes in the cell compositions and their individual gene expression profiles during lung rejection. They found that the Mzb1-expressing plasma cell population in the lungs with bronchiolitis obliterans syndrome increased more than the others. Also, CD14-expressing monocytes and PDGFRA-expressing fibroblasts were increased. They also performed pseudo-time and trajectory analysis, based on which they found that a Bhlhe41, Cxcr3, ITGB1-triple positive-B cell subset plays as the progenitor pool for Mzb1+ PCs, which results in IgG2c expression and production in the grafts with bronchiolitis obliterans syndrome. 

Researchers have investigated the generation, maintenance, and function of human lung tissue-resident memory T cells in transplanted lung samples [137]. They dynamically tracked the donor and recipient T cells. They realized that the donor T cells remain in the transplanted lungs and highly express their markers, including CD69, CD103, and CD49a; however, the lung-infiltrating recipient T cells acquire the phenotypes over months. By using scRNA-seq, they identified two donor T cell subsets with different marker gene expressions; however, recipient T cells were composed of non-tissue-resident memory T cells and tissue-resident memory T cells-like subpopulations, suggesting de novo TRM generation. 

Researchers have argued that RNA sequencing analysis indicates that lung disease after severe and prolonged SARS-CoV-2 infection shows pathological and molecular features similar to the ones in pulmonary fibrosis requiring transplantation [138]. This finding suggests that lung transplantation might be necessary for these affected individuals. They also report successful lung transplantation for these patients. 

**Figure 4:**
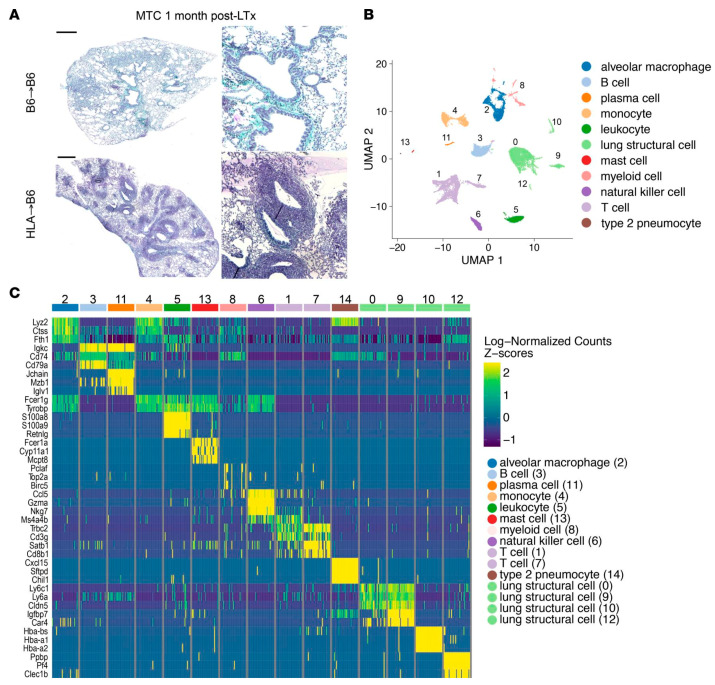
(**A**) Histology sections of control (B6→B6) and bronchiolitis obliterans syndrome (HLA→B6) mouse lung graft one month post-transplantation. Scale bars, 500 μm. Zoomed views are shown on the right. (**B**) UMAP plots of 11 cell populations. (**C**) Heatmap illustrating most upregulated genes in each cell cluster. The figure is taken with permission from [136] under Creative Commons Attribution 4.0 International License (https://creativecommons.org/licenses/by/4.0/), accessed on 1 February 2024).

Figure 4 illustrates the single-cell analysis results of a mouse lung graft in which 11 cell populations were found. In Table 5, some key findings in lung transplantation based on scRNA-seq are listed. Also, related biomarkers in lung transplantation are listed in Table 6.

### 3.4. Liver Transplantation

**Figure 5:**
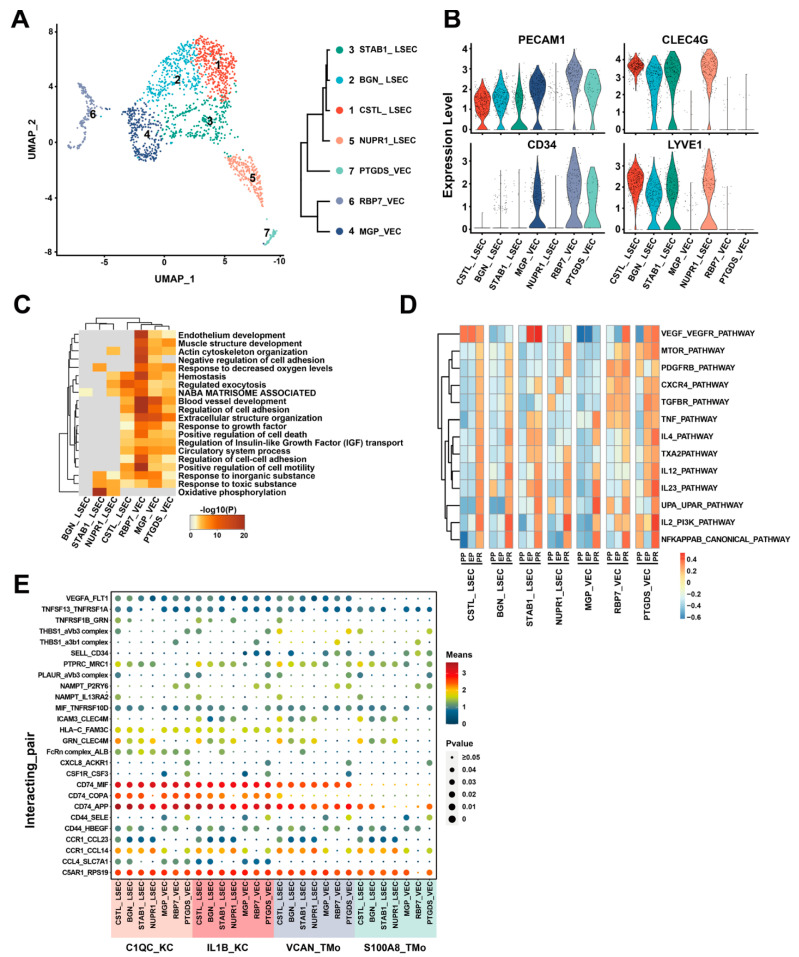
Single-cell RNA-sequencing of endothelial cells after liver transplantation. (**A**) UMAP plot presenting seven endothelial cell clusters (**left**). Dendrogram of the seven clusters (**right**). (**B**) Violin plots demonstrating the normalized expression of PECAM1, CLEC4G, CD34, and LYVE1 genes. (**C**) Gene Ontology enrichment analysis results. (**D**) Gene set variation analysis identifying the pathways. Different colors stand for different activation scores. (**E**) Cell-cell interaction analysis between mononuclear phagocyte clusters and different endothelial cell clusters. The circle size stands for the level of *p*-value. Colors stand for different mean values. The figure is taken from [153] with permission under a Creative Commons Attribution 4.0 International License (http://creativecommons.org/licenses/by/4.0/, accessed on 1 February 2024).

Liver transplantation is considered a treatment strategy for patients with hepatocellular carcinoma, and scRNA-seq is a promising technique for detecting associated problems. By using flow cytometry, some cell subpopulations in liver samples can be detected [150,151]; however, unbiased scRNA-seq shows that the heterogeneous cell population in the liver is much wider than what is detected by the flow cytometry and consists of numerous subpopulations [152]. Most importantly, researchers used this technology (10× Genomics) to obtain an unbiased and comprehensive liver transplant cell atlas by collecting liver tissue samples pre-procurement, post-preservation, and two hours post-reperfusion [153]. They identified different cell subgroups, their transcriptome changes, and the interactions between them during liver transplantation. The results of this work can be used to realize the cellular and molecular mechanism of graft ischemia–reperfusion injury during liver transplantation. See Figure 5 for scRNA-seq of endothelial cells in liver grafts.

Another research group used single-cell analysis on transplanted liver samples and detected a subset of CSF3+ Kupffer cells that is related to the injuries associated with graft transplantation injury [154]. They also found higher levels of dendritic cells and CD8+ T cells in the fatty liver donors. In a preprint, authors classified the liver cells into 14 cell types and 29 subpopulations with different cell states [155]. They claimed to have found pathogenic cellular modules associated with early allograft dysfunction, consisting of mucosal-associated invariant T cells, granzyme B, granzyme K, natural killer cells, and S100A12 neutrophils.

Recently, based on scRNA-seq, the complex landscapes of organogenesis containing liver development and decidualization were analyzed [156]. In this study, scRNA-seq and cytometry by time of flight (CyTOF) were used to uncover the cell states and sources involved in liver graft remodeling. They also used transcriptome data to show the interplay among hepatocytes and macrophages. The transcriptomic data they obtained revealed that the complexity of the metabolic remodeling of the transplanted liver is a complex task in which a regulatory network of ligands and receptors among macrophages and hepatocytes is involved.

Subpopulations of various hepatic cell types containing macrophages, epithelial progenitor cells, and myofibroblasts and their behavior were uncovered with the application of scRNA-seq on human and zebrafish livers [157]. They applied scRNA-seq on single cells obtained from the livers of 18-month-old male zebrafish to uncover the transcriptional profiles of the cell types available in the liver and to use them as tools to understand liver function and diseases. They determined the similarities between the transcriptomic data of adult zebrafish liver and the human liver single-cell transcriptome. The next step of this study could be on transplanted livers in zebrafish.

Key findings and biomarkers of scRNA-seq in liver transplantation are listed in Table 7 and Table 8, respectively.

### 3.5. Other Transplants

The scRNA-seq technique is also used in organ transplant recipients with squamous cell carcinoma. This method is combined with T-cell receptor sequencing to define the T-cell prospect in cutaneous squamous cell carcinoma [172]. This method is used to find tumor-infiltrating lymphocyte phenotype in squamous cell carcinoma from immune-competent and immune-suppressed patients. CD8+ T cells from the samples were sequenced to distinguish various T cell populations. Also, the T cell immune response was characterized by sequencing the α and β CDR3 regions.

## 4. Stem Cell Transplantation

Stem cell transplantation is considered a promising therapeutic method for various diseases, including blood diseases, immune system diseases, neurodegeneration, and cancer [173,174,175]. However, to be considered safe and reliable, a clear understanding of the cell behavior after transplantation is needed. Stem cells have shown promise in treating lung diseases. Pulmonary fibrosis (PF) is an example of a chronic lung disease for which treatment adipose-derived mesenchymal stem cells (ADSCs) are considered candidates. To understand the underlying mechanisms, the interaction between ADSCs and lung cells was studied at the single-cell level [176]. Using scRNA-seq, the authors realized that ADSC treatment changed both the transcriptomic profile and the composition of the lung cells, especially macrophages. They also identified potential signaling pathways, such as NGR, ANNEXIN, HGF, and PERIOSTIN. They found that the ADSCs increased the Trem2+ anti-inflammatory lung macrophages. They also decreased inflammation and fibrosis in the lung. 

The umbilical cord blood (UCB) transplant is considered a promising therapeutic option for multiple diseases (e.g., blood cancers, myeloproliferative disorders, and genetic diseases). Single-cell analysis has revealed the cellular heterogeneity in the nucleated cells in UCB [177]. The authors reported 12 major cell types with multiple subpopulations.

Researchers extracted the single-cell full-length transcriptome data to construct an isoform-based transcriptional atlas of the murine endothelial-to-hematopoietic stem cell transition [178]. They used the obtained atlas to identify the hemogenic signature isoforms and the alternative splicing events. The results are crucially important because the transcribed mRNAs typically undergo alternative splicing, which affects the transcript isoforms and results in different proteins. 

Allogeneic hematopoietic stem cell transplantation is considered a treatment method for malignant hematological diseases. Tracking of T-cells in transplantation is important and may uncover information about the graft-versus-leukemia effect. Researchers used single-cell RNA sequencing to extract the transcriptomic data of ~35,000 single T cells in the blood of 14 samples before and after transplantation [179]. They reported a huge drop in unique T-cell clones post-transplantation compared with the donor samples.

To answer the need for cell replacement in diabetes, human pluripotent stem cells differentiated into insulin-secreting β cells in islet organoids can be used. However, the behavior of these cells in vitro compared with native adult β cells is different. Single-cell transcriptomic profiling can detect the transcriptomic changes in these cells. By using this method, researchers have realized that transplanted insulin-secreting β cells show a behavior closer to the adult β cells [180]. They showed an increase in the insulin and IAPP protein secretions after transplantation. The obtained results of this study provide a wealth of information about the human islet cell maturation, as well as the maturation of the insulin-secreting β cells.

**Figure 6:**
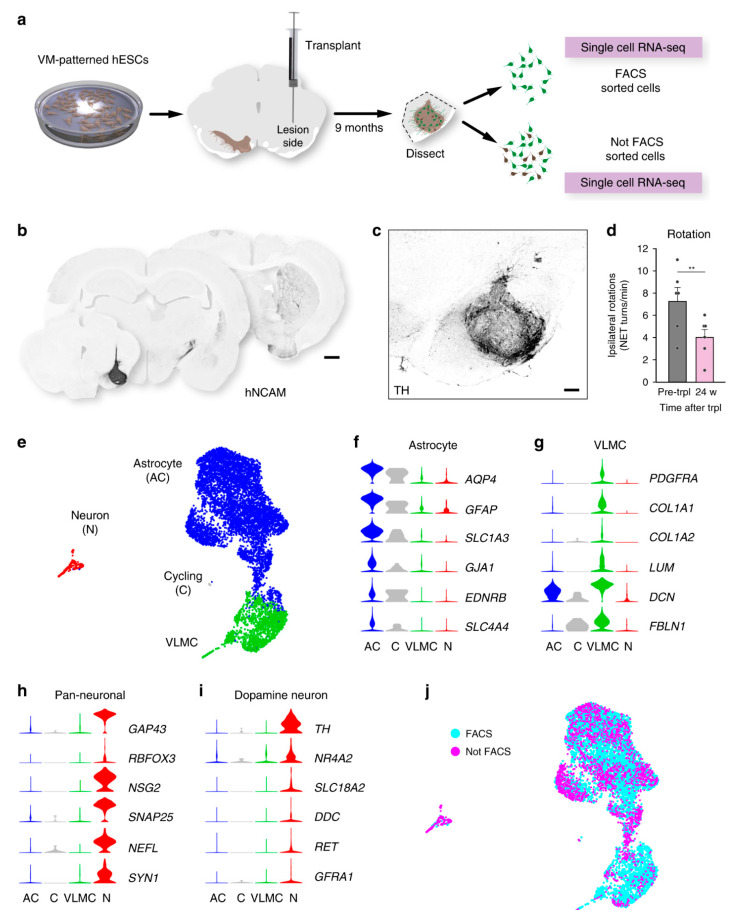
The scRNA-seq technique and histological validation of transplanted cells into the midbrain. (**a**) Schematic of experiments. VM-patterned hESCs: ventral midbrain-patterned human embryonic stem cells. (**b**) Growth of hNCAM fiber from hESC-derived graft. (**c**) Immunohistochemistry. (**d**) Drug-induced rotation test that shows functional recovery in rats after transplantation (*n* = 6 rats; mean ± SEM; ** *p* < 0.01; compared to post-lesion; two-tailed paired *t*-test). (**e**) UMAP plots of 7875 analyzed cells after transplantation. (**f**–**i**) Expression level for each cluster of biomarkers. (**j**) UMAP of transplanted cells for cells isolated by FACS (blue circles, *n* = 5958) and not by FACS (magenta circles, *n* = 1917). Scale bars, 1 mm (**b**); 200 µM (**c**). The figure is taken with permission from [181] under a Creative Commons Attribution 4.0 International License (http://creativecommons.org/licenses/by/4.0/, accessed on 1 February 2024).

Stem cells have been transplanted for brain disorder treatment as well. The cells to be transplanted are usually obtained from the fetal brain tissue or the stem cells. After transplantation, the dopamine neurons are rare, determining the identity of other cell types. Researchers used single-cell RNA sequencing on a rat model to characterize the grafts from the human embryonic stem cells and fetal tissue [181]. They found a high level of neurons and astrocytes in both cases, while they found an additional perivascular-like cell type in the stem cell-derived grafts. Figure 6 illustrates the performed scRNA-seq analysis, as well as the histological validation of the transplanted cells into the midbrain.

Muscle stem cells maintain their regenerative capabilities after transplantation into recipient hosts [182,183]. However, primary myoblasts do not show the ability to engraft and proliferate after transplantation. The scRNA-seq technique is used to uncover the transcriptional state and developmental dynamic trajectories of injured muscle stem cells and primary myoblasts [132]. Researchers aligned the obtained transcriptomes of muscle stem cells derived from homeostatic and injured muscles, as well as primary myoblasts, along a pseudo-temporal single trajectory to order them (unsupervised). This method allowed them to describe the progression of the differentiation process at the single-cell level. They found two clusters (i.e., close-to-quiescence and early-activation clusters) with partially overlapping transcriptomes. They used bioinformatic techniques to recognize the difference between the two and place them in distinct space-time pathways. 

Researchers have used scRNA-seq to provide the transcriptional landscape of human hematopoietic progenitors at the single-cell level [133]. They then showed that the CD38 antigen, which is usually considered the biomarker to negatively enrich the primitive progenitors for transplantation, is not a good choice. By showing the biological relevance of the CD164 gene in early hematopoiesis, they suggested using this marker instead in clinical transplantation and gene therapy. In another work, where scRNA-seq was used to classify transplanted hematopoietic stem cell identities at various differentiation stages, researchers found that the branching of hematopoietic lineage differentiation in adult marrow occurs at the transcriptional hematopoietic stem cell and transcriptional multipotent progenitor stages [184]. However, they found that the majority of transplanted hematopoietic stem cells committed to transcriptional multipotent progenitors. The proliferation of the donor-derived hematopoietic stem cells surviving after transplantation is accompanied by gradually decreasing the hematopoietic stem cell population. However, a balance between proliferation, differentiation, and stem cell maintenance maintains the cell functions at the bulk level. 

To identify the role of tissue-resident memory T cells in the host defense system, samples from allogeneic hematopoietic stem cell transplanted patients were used, and the interindividual variation in host skin tissue T cell maintenance was studied [185]. Long-term persistence of host skin T cells that is reported not to be consistent with the chronic graft-versus-host disease development was seen in a group of patients. 

The scRNA-seq technique was used to show that both fetal ventral midbrain and human embryonic stem cells-derived dopamine progenitors increase neurons and astrocytes after grafting [181]. Oligodendrocytes were present in fetal cell grafts; however, a cell type not known as a part of neural grafts was seen in grafts of human embryonic stem cells-derived ventral midbrain-patterned progenitors. The results of scRNA-seq of the cells before transplantation and at the time of transplantation indicated that genes associated with vascular leptomeningeal cells and progenitors were expressed. This finding suggests the potential of human embryonic stem cell-derived progenitors for generating both neural and perivascular cell types at this time. The scRNA-seq results suggest that future studies should investigate the contribution of various cell types to graft function. These studies can open the window to understanding the role of vascular leptomeningeal cells, astrocytes, and oligodendrocytes in the behavior of the graft.

The scRNA-seq technique was used to study the dynamic gene expression profile during limbal stem cell differentiation [186]. Expression heterogeneities among subgroups of the differentiated cells were detected. Epithelial–mesenchymal transition during the differentiation process, which may result in the generation of untargeted cells, was reported. Pseudo-time trajectory showed changes in transcriptions and signs of commitment for limbal stem cells and their progeny. The new markers found for limbal stem cells in this study need further work to identify their origin and accuracies.

Table 9 and Table 10 show some key findings and biomarkers of single-cell analysis in stem cell transplantation, respectively. 

## 5. Tumor Transplantation

Another application area in the field of transplantation is where it is used to study cancer therapeutic methods in vivo. A tumor is transplanted in an animal model, and then its behavior and response to cancer drugs are studied. However, single-cell analysis shows different immune landscapes in transplant and primary tumors and distinct responses to immunotherapy. Kirsch, from Duke University, and his coworkers showed that PD-1 blockade and radiotherapy can be used to cure transplant sarcomas; however, this protocol does not work in autochthonous sarcomas [194]. They found differences in immune landscapes of tumor-infiltrating immune cells from transplanted and primary tumors. 

In most preclinical studies in cancer biology, researchers transplant tumors to study them in vivo. With the help of single-cell analysis, researchers have shown that although transplant sarcomas can be treated by programmed cell death-1 (PD-1) blockade and radiotherapy, this treatment cannot cure autochthonous tumors. This group used scRNA-seq and mass cytometry to study tumor-infiltrating immune cells from transplanted and primary tumors before and after radiation therapy and anti-PD-1 immunotherapy and found different immune profiles. They found that transplanted tumors are enriched for activated CD8+ T cells and PD-L1+ macrophages and concluded that PD-1 blockade and radiotherapy may be good treatments for patients with a sarcoma immune phenotype similar to those transplanted tumors [194].

**Figure 7:**
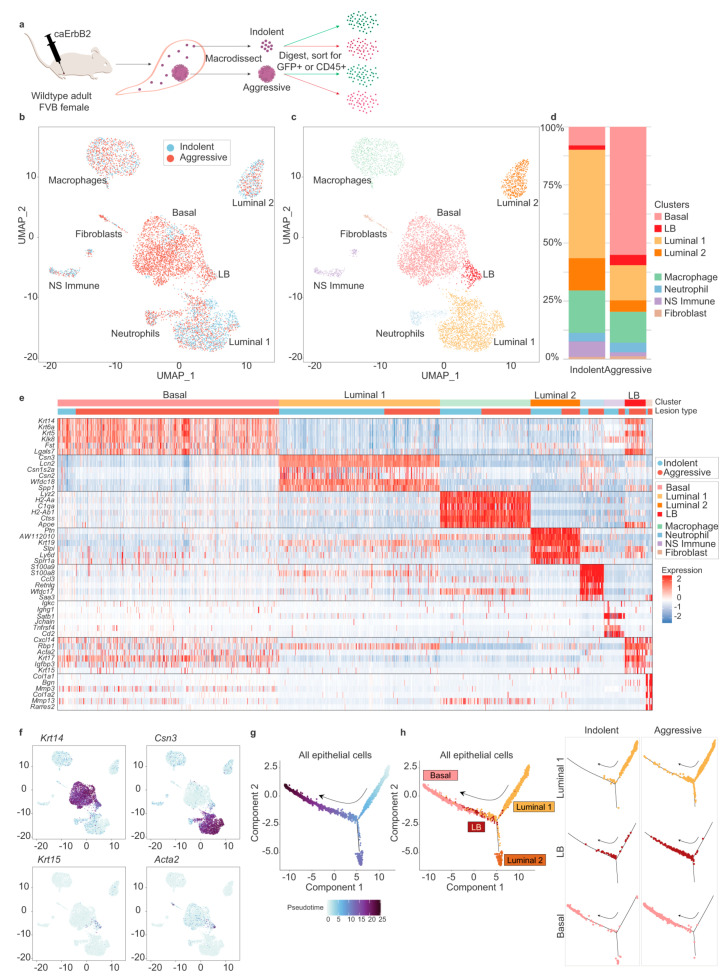
The scRNA-seq experiments on breast tumor transplanted mice. (**a**) Schematic of scRNA-seq experiments. Indolent tumor cells, indolent immune cells, aggressive tumor cells, and aggressive immune cells were collected from 13 pooled mice. (**b**–**d**) UMAP plots of the single-cell data. Clusters proportions are depicted in (**d**). (**e**) Heatmap of highly differentially expressed genes for each cluster. (**f**) UMAP plots of all tumor cells, with colors indicating expression of KRT14, Csn3, KRT15, and Acta2 for basal-like cells, Luminal 1 cells, and Luminal-Basal cells, respectively. Trajectory plot of all tumor cells, with colors indicating pseudo-time value (**g**) and cluster ((**h**), **left panel**). ((**h**), **right panel**) Trajectory plots of Luminal 1, LB, and basal cells are shown. The arrows depict increasing pseudo-time value. The figure is taken with permission from [195] under a Creative Commons Attribution 4.0 International License (http://creativecommons.org/licenses/by/4.0/, accessed on 1 February 2024).

The scRNA-seq technique applied to transplanted breast cancer tumors in mice shows that the aggressive tumor niche is determined by a basal-like population and mixed-lineage cancer cells [195]. The analysis showed two luminal-like populations (i.e., major Luminal 1 and minor Luminal 2). Figure 7 shows the scRNA-seq experiments and the obtained results.

See Table 11 and Table 12 for examples of key findings in scRNA-seq applied to tumor transplantation and the related biomarkers, respectively.

## 6. Conclusions

Single-cell RNA sequencing is an exciting new technology with the capability of driving significant progress in various bio-disciplines. It allows researchers to examine individual cells and uncover the molecular dynamics within them, revolutionizing the way biological processes can be studied. One of the important fields in which scRNA-seq has made fundamental advancements is transplantation. 

Transplantation is the care gold standard for end-stage organ diseases; however, not all transplantations are successful. The most frequent complication of transplantation is allograft rejection. Currently, the diagnosis of these complications in the clinical setting needs biopsies obtained from the patients. However, traditionally found biomarkers are not fully reliable for detecting rejection.

The scRNA-seq technique uncovers cell heterogeneity, cell states, and graft complications in solid transplantation and stem cell transplantation. It is also used in studying transplanted cancer cells. The scRNA-seq technique can provide a comprehensive single-cell atlas of gene expression profiles in acute rejection and transplant complications. It uncovers the contribution of T cells and natural killer cells, as well as the association of various subsets of macrophages, including infiltrating (m3 and m4) and resident macrophages (m1 and m2), in graft rejection. Major findings include cell diversity in grafts, gene expression profile variation during graft rejection, identifying novel biomarkers, uncovering macrophage polarization, and immune profile landscapes. 

The obtained large and high-dimensional data from single-cell analysis needs computational data processing and analysis. Machine learning methods are employed to develop analysis pipelines and predictive models toward this goal. In the future, more advanced machine learning methods will further contribute to the development of the field.

## Figures and Tables

**Table 1:** Comparison of some of the common scRNA-seq methods.

Methods	Advantages	Disadvantages	References
Smart-seq Smart-seq 2 Smart-seq 3	Full-length transcript coverage, high sensitivity, low technical noise	Low throughput, requires manual cell isolation, high cost per cell	[75,78,79]
Drop-seq	High throughput, low cost per cell, large-scale parallel processing, droplet-based microfluidics	Limited coverage of full-length transcripts, low sensitivity, high technical noise	[60]
In-Drop	High throughput, low cost per cell, large-scale parallel processing, droplet-based microfluidics, efficient for analyzing limited cell numbers compared with Drop-seq	Limited coverage of full-length transcripts, low sensitivity, high technical noise	[72]
10× Genomics Chromium	High throughput, easy to use, compatible with a wide range of samples, droplet-based microfluidics	Limited coverage of full-length transcripts (if paired with long reads technologies such as nanopore sequencing [87,88]), lower sensitivity compared with Smart-seq3	[89]
Seq-well	High throughput, low cost, easy scalability, ability to multiplex samples, based on microfluidics	Limited coverage of full-length transcripts, lower sensitivity compared with Smart-seq3, Needs manual pipetting.	[74]
sci-RNA-seq	High throughput, high sensitivity, full-length transcript coverage	More technically challenging than some other methods, requires specialized equipment	[80]
MATQ-seq	High throughput, low technical noise, high sensitivity, full-length transcript coverage	More technically challenging than some other methods. Needs manual pipetting.	[81]
Nanopore Sequencing	Long reads, portable	Relatively higher error rates	[87,90]

**Table 2:** Examples of key findings in applying scRNA-Seq on heart transplantation.

Key Findings	Methods/Technologies	Donor/Recipient Species	References
**Cellular diversity:** The scRNA-seq technique has been used to reveal the cellular heterogeneity in the heart, including immune cells, fibroblasts, endothelial cells, macrophages, and cardiomyocytes.	10× Genomics Chromium	Mice, human, pig	[91,96,97]
**Immune cell populations in rejection:** The scRNA-seq technique has identified various immune cell subsets involved in graft rejection, such as T cells, B cells, natural killer cells, and macrophages. Transcriptional profiles and functional states of these cells during rejection are considered key findings.	inDrop Microfluidics, 10× Genomics Chromium	Mice, human	[7,98,99]
**Gene expression profile variations during rejection:** Specific gene expression changes during rejection in various cell types (e.g., interferon-stimulated genes upregulation in T cells, proinflammatory pathways activation in macrophages, and upregulation of extracellular matrix genes in fibroblasts) are identified.	10× Genomics Chromium	Mice, human	[7,100,101,102]
**Potential therapeutic target recognition:** The scRNA-seq technique has been used to identify novel potential targets for therapeutic purposes in heart transplantation (e.g., IL-18 signaling and Hif1a inhibiting in T cells and CXCR6 in natural killer cells and T cells).	10× Genomics Chromium	Mice, human	[91,103,104]
**Biomarker discovery:** The scRNA-seq has been used to find gene expression signatures that can be considered biomarkers for predicting organ rejection or checking the responses to immunosuppressive therapy.	10× Genomics Chromium	Mice, human	[91,105]

**Table 3:** Examples of key findings in applying scRNA-Seq on kidney transplantation.

Key Findings	Methods/Technologies	Donor/Recipient Species	References
**Cellular diversity:** The scRNA-seq technique has been used to reveal the cellular heterogeneity in the kidney, including immune cells, macrophages, IFNg, myeloid, and T cell subclusters. These heterogeneities represent distinct signatures that have different roles in allograft loss.	10× Genomics	Mice, human	[5,112,113,118,129,130]
**Immune cell populations in rejection:** The scRNA-seq technique has identified various immune cell subsets involved in graft rejection, such as T cells, B cells, neutrophils, myeloid cells, dendritic cells, stromal cells, and macrophages. Transcriptional profiles and functional states of these cells during rejection are considered key findings.	10× Genomics	Mice, human	[113,115,116,129]
**Gene expression profile variations during rejection:** Altering myeloid cell differentiation and their behavior based on upregulating expressions of ribosomal protein genes may affect the implant.	10× Genomics	Mice, human	[112,116,118,127]
**Potential therapeutic target recognition:** The scRNA-seq technique has been used to identify novel potential targets for therapeutic purposes in kidney transplantation.	10× Genomics	Mice, human	[5,111,116,127,131]
**Biomarker discovery:** The scRNA-seq technique has been used to find gene expression signatures that can be considered biomarkers for predicting organ rejection or checking the responses to immunosuppressive therapy.	10× Genomics,	Mice	[17,132,133]
**Uncover novel cell types:** The scRNA-seq technique assists in finding novel cell types and statuses without any bias or RNA degradation.	10× Genomics,	Human, mice	[113,129,131,134]
Cells (e.g., some glomerular endothelial cells) from the recipient or donor (e.g., the renal architecture) may represent endothelial chimerism.	10× Genomics	Human	[116]
The scRNA-seq technique shows that leukocyte populations mostly express sex-linked genes from recipients, which may be linked to immune cell infiltration. For example, natural killer cells and monocytes are involved in kidney rejection,	10× Genomics	Human	[116]

**Table 4:** Examples of important biomarkers of kidney transplantation complications.

Gene Biomarkers	Methods/Technologies	Donor/Receptor	References
IFNg, GSVA, and DEGs	10× Genomics	Mice	[110]
RTK and Axl	10× Genomics	Mice	[118]
PDGF, ECM, and TGF-β	10× Genomics	Human	[113]
Nphs2CremT/mG, SclCremT/mG, Cdh16CremT/mG, AQP3, and HSD11B2	Droplet-based	Mice	[131]
CXCL10	10× Genomics	Human	[116]
TRDC, CD4, CD8A, KLRK1, ITGAX, CD19, and CD14	10× Genomics	Human	[111]
PGs, *GGT5*, and *EMILIN1*	10× Genomics, Drop-seq	Human	[129]
CD3E, MS4A1, SDC1 (CD138), and TPSAB1	Droplet Microfluidics	Human	[112]
ALDOB, GATM, GPX35, JUN, VIM, HSP, ALDOB, GPX3, GATM, CTGF, CXCL12. CAV1, COL4A1/A2, VIM, COL4A2, and VWF	Droplet Microfluidics	Human	[134]
TFAIP3, CXCR4, ZFP36, S100A8, S100A9, CXCL8, FOS, MTND6, HLA-DQA2, MT-ND6, CXCL8, NFKBIA, CD69, CD83, and HLA-DQA2			[127]
CD19 and CCR6		Human, mice	[115]
CD16+, CD162, ABCA1, APOE, PDE3A, IGKC, LGMN, iCD83, FCGR3A, CD16, and FCN1	Droplet Microfluidics	Human	[5]

**Table 5:** Examples of key findings of applying scRNA-seq to lung transplantation.

Key Findings	Methods/Technologies	Donor/Recipient Species	References
**Cellular diversity:** The scRNA-seq technique is reported to identify cell populations associated with bronchiolitis obliterans syndrome.	10× Genomics	Mice, human	[136,139,140,141,142,143,144]
**Immune cell populations in rejection:** In acute cellular rejection, a clonal population of cytotoxic and effector CD8+ T cells exist in the transplanted lung and remain after treatment.	10× Genomics	Mice, human	[136,139,140,145]
**Potential therapeutic target recognition:** A subgroup of innate B-1 cells may contribute to autoimmunity in bronchiolitis obliterans syndrome, which represents a potential therapeutic method.	10× Genomics	Mice, human	[136,139,140]
**Biomarker discovery:** The scRNA-seq technique has been used to find gene expression signatures that can be considered biomarkers for predicting organ rejection or checking the responses to immunosuppressive therapy.	10× Genomics	Mice, human	[136,143]
**Macrophage polarization:** Macrophages are found to be heterogeneous cell populations, which upon activation, polarize into various phenotypes. After transplantation, tissue-resident macrophages quickly change their gene expression profile into that of the host organ markers.	10× Genomics	Mice, human	[146,147]
**Uncovering novel cell types:** For example, additional endothelial and lymphatic cell populations, megakaryocytes, innate lymphoid cells, and mesothelial cells have been identified in mice.	10× Genomics	Mice	[148]

**Table 6:** Examples of important biomarkers of lung transplantation complications.

References	Methods/Technologies	Cells	Donor/Receptor	Gene Biomarkers
[149]	10× Genomics	Emphysema, cystic fibrosis, sarcoidosis	Human	CD6914, CD103, CD69+, CD137+, CD69+ and/or OX40+
[136]	10× Genomics	COPD, CTD-ILD	Human, mouse	Bhlhe41, Zbtb20, Cxcr3, Itgb1, CD19, CD43, CD5, Xbp1, Sdc1, Mzb1, Irf4, Ighm,
[141]	10× Genomics	Bronchopulmonary dysplasia	Mouse, human	Epcam, Pecam1, Ptprc, Col1a1, Msln,
[142]	10× Genomics	Adenocarcinoma, endobronchial carcinoid, LLL endobronchial typical carcinoid,	Mouse, human	EPCAM, CLDN5, COL1A2, PTPRC, CD31, CD45, KRT5, MKI67, SERPINB3, C20orf85, CLDN5, MYC, ACKR1, ACKR1, GJA5, CCL21, CLDN5 with DAPI, COL1A2, GPC3, Slc7a10, SERPINF1, Pi16, ASPN, COX4I2, COL1A2, APOE, GPR183, Slc7a10,
[144]	RNeasy Plus Mini kit (QIA GEN)	Fibrotic lung disease, idiopathic pulmonary fibrosis, systemic sclerosis-associated interstitial lung disease, interstitial pneumonitis, pneumoconiosis	Mouse, human	CD206, CD169

**Table 7:** Examples of key findings in applying scRNA-seq on liver transplantation.

Key Findings	Methods/Technologies	Donor/Recipient Species	References
**Cellular diversity:** The scRNA-seq technique has been used to uncover the cellular heterogeneity in the liver, including immune cells, macrophages, IFNg, myeloid, and T cell subclusters. These heterogeneities highlight signatures with different roles in allograft complications.	10× Genomics	Human, rat	[155,158,159,160,161,162,163,164,165]
**Immune cell populations in rejection:** The scRNA-seq technique has identified various immune cell subsets involved in graft rejection, such as T cells, B cells, neutrophils, myeloid cells, dendritic cells, stromal cells, macrophages, and their transcriptional profiles and functional states during organ rejection. Some cell populations, including IL-7R+CD4+ T cell, and CRTAM+CD8+ T cell, are shown to be reduced in the transplanted liver.	10× Genomics	Human, mouse	
**Gene expression profile variations during rejection:** Gene expression variation of B cells in bronchiolitis obliterans syndrome is uncovered.	10× Genomics	Mice	[136]
**Potential therapeutic target recognition:** The scRNA-seq technique has been used to identify novel potential targets for therapeutic purposes in liver transplantation. For example, it helps to understand the heterogeneity of LDLR+MDSC and CTLA4+CD8+ T, especially CD4+CD8+FOXP3 T cells, which may result in finding innovative therapeutic methods.	10× Genomics	Human	[158]
**Biomarker discovery:** Machine learning and scRNA-seq have helped in identifying novel biomarkers.	10× Genomics	Human	[166]
Macrophage polarization	10× Genomics	Human	[152,167]
Uncovering novel cell types	10× Genomics	Human, rat	[155,158,162,163,165]

**Table 8:** Examples of important biomarkers of liver transplantation complications.

References	Sequencing Method	Cells	Donor/Receptor	Gene Biomarkers
[158]	10× Genomics	Liver cirrhosis, hepatocellular carcinoma	human	LDLR, GZMB, GZMA, GZMB, GZMH, NKG7, GZMK, DUSP4, and COTL1
[159]	10× Genomics Chromium	Hepatocellular carcinoma, cirrhosis of the liver	human	TCRs, BCRs, CD3D, KLRF1, CD79A, IGHG1, CD177, CD68, PECAM1, KRT7. CD4+ T cell lineages, CD4+, (Tem, GZMK), CD4+ (CCR7, LEF1), CD4+, (MAIT, SLC4A10), (MKI67)
[155]	10× Genomics	Hepatocellular carcinoma and primary sclerosing cholangitis disease	Human, rat	S100A12, LTF, PRTN3
[152]	10× Genomics	Liver cancer	Human, mouse	KRT19 (CK19), EPCAM, FXDY2, CLDN4, CLDN10, SOX9, MMP7, CXCL1, CFTR, TFF2, KRT7 (CK7), CD24
[160]	10× Genomics	Para-tumor liver tissue, cirrhotic	human	EPCAM, SOX9, AFP, KRT7, S100A6, S100A11, ALB, PCK1, FGG, FGA, TTR, EBPB, APOB, CYP2E1, APOE
[161]	Seven Bridges Genomics	Cholangiosepsis	human	CD15, CD68, CD3, CD8, CD20, FCGR3B, CD68, CD3E, CD4, CD8A, Tregs, FOXP3, NKG7, FLT3, CD24, CD79A, JCHAIN, ALB, FLT1, KRT19, IFITM2, CSF3R, FPR1, FCGR3B, VNN2, G0S2, CXCR2, SOD2. CXCR2, CXCR4, CD83, CCRL2, CCL3, CCL4, ICAM1, VEGFA, CST3, CTSB, MS4A7, MARCH1, CD68, MAFB, CD163, VCAN, CSF1R, LYZ, VCAN, S100A8, S100A9, S100A12, MNDA
[162]	10× Genomics	Chronic hepatitis B (CHB), HBV-associated liver cirrhosis (LC) patients	human	CD3D, KLRF1, CD19, SDC1, CD14, FCGR3A
[155]	10× Genomics	Chronic liver disease	Mice	S100A6, Ccl2, Cxcl1, Cxcl12, Col1a2, Col3a1, Col5a2
[163]	10× Genomics	Human liver cirrhosis	Human, mice	MNDA, CD9, TIMD4
[164]	CEL-seq2	Colorectal cancer metastasis or cholangiocarcinoma	Human, mouse	AKR1B10, MKI67, PCNA, ALB, HP, HNF4A, ASGR1, PROX1, KRT19, CFTR, ASGR1 plus ALB, CXCL8 plus MMP7, PECAM1, CLEC4G, CD34, CLEC4M and FLT1
[165]	10× Chromium Smart- seq2	Hepatocellular carcinoma (HCC)	Human	CD14, CD2, CD3D, CD4, CD68, LYZ, MS4A1
[168]	10× Chromium	Nonalcoholic steatohepatitis (NASH), HCV	Human	CD45, CD31, CD68, CD146, SSC-A, PDPN, CCL21, LYVE1, FLT4, PROX1
[169]	10× Chromium Smart- seq2	Solitary colorectal metastasis	Mouse	Mki67, Col1a1+, NGFR, Adamtsl2
[170]	Droplet-based sequencing and data analysis, 1× Genomics	Cholangiocarcinoma	Mouse	CD68, CK-19, MHCII, MHCI, CD45, CD11b, Ly6G, Ly6C, CD19, CD115, B220, TER-119, Tim4, NK1.1, MERTK, CD8a, CD3e, TCRb, CD206, Lgals3, CD11c, CX3CR1, CCR2, F4/80, CD14, CD64
[171]	DNBSEQ-G400RS (MGI Tech)	Cholangiocarcinoma	Mouse	Alb, Apoa1, Ass1, Spp1, Sox9

**Table 9:** Examples of key findings in applying scRNA-Seq on stem cell transplantation.

Key Findings	Methods/Technologies	Donor/Recipient Species	References
**Cellular diversity**	Illumina Hiseq platform (Novogene), 10× Genomics	mouse	[184,187,188]
**Cell populations:** A neutrophil progenitor population that highly expresses S100A gene family members is detected in transplanted hematopoietic stem cells. Combined with FACS, scRNA-seq can ensure cellular purity in samples. Evaluating the ability of the bone marrow-mesenchymal to differentiate into subpopulations is possible.	10× Genomics Chromium	mouse, human	[189,190]
**Gene expression profile variations**	Illumina Hiseq platform (Novogene), 10× Genomics Chromium	mouse	[180,184,187]
**Potential therapeutic target recognition:** The scRNA-seq technique identifies therapeutic targets for osteosarcoma.	10× Genomics	mouse, human	[191]
**Uncover novel cell types:** The scRNA-seq technique assists in uncovering novel cell types.	10× Genomics	mouse, human	[188]

**Table 10:** Examples of important biomarkers of stem cell transplantation complications.

References	Methods/Technologies	Notes	Donor/Receptor	Gene Biomarkers
[187]	10× Genomics	two iliac cristae, two tibiae and two femora	mice	CD41, CD150
[184]	Illumina Hiseq platform (Novogene)	hematopoietic system	mouse, rat	Lin-Sca1+Kit+CD34-Flk2- Lin-Sca1+Kit+CD34-CD150+CD41- Lin-Sca1+Kit+CD34-CD150-CD41- CD201+CD150+CD48-CD45+ CD201+CD150+CD48-CD45+Sca1+Kit+
[180]	10× Genomics Chromium	Diabetes	mouse	MAFA, FAM159B, NAA20
[188]	10× Genomics	acute myeloid leukemia (AML) patients	mouse, human	CCR10, TNFRSF18, GZMK, CD8A, TNFRSF18, SIGLEC7, GNLY, LGALS3, CCR10, CD4, CLEC4C, PF4, PTCRA, CD8B, ID3, CD79A
[192]	10× Genomics	hematological malignancies,	human	CD3D, CD4, IL7R, CCR7, CCR6, CCL5, TBX21, FOXP3, CD8A, CD8B, CXCR6, RORC, CD69, IFIT3, GZMH, TRGC1, XCL1, XCL2, IL1R1, KIT, IFNG, FCGR3A
[193]	10× Genomics	Wolfram syndrome (WS)	mouse, human	SPINK1, ID3, NKX2-2, MAFB, NKX6-1, NKX2-2, GCK, ISL1, PDX1

**Table 11:** Examples of key findings in applying scRNA-seq on tumor transplantation.

Key Findings	Methods/Technologies	Donor/Recipient Species	References
**Cellular diversity:** The intra-individual, interindividual, spatial, functional, and genomic heterogeneity in melanoma cells, as well as tumor factors affecting the microenvironment (e.g., tumor-infiltrating immune cells, tumor-associated fibroblasts, and endothelial cells), are identified.	10× Genomics	human	[196,197,198]
**Key factors:** TNF receptor-related factor 3 (Traf3) is found to be significantly mutated in murine intrahepatic cholangiocarcinoma. In human intrahepatic cholangiocarcinoma, an inverse correlation between Traf3 and NF-κB-inducing kinase expression is reported. NF-κB-inducing kinase inhibition damps the growth of intrahepatic cholangiocarcinoma.	DNBSEQ-G400RS (MGI Tech), 10× Genomics	mouse	[171,196,197]
**Gene expression profile variations:** The scRNA-seq on the liver had identified mostly convergent gene expression alterations when primary biliary cholangitis and primary sclerosing cholangitis were compared to normal controls. Genes expressed by one cell type (e.g., CAFs) may affect the proportion of other cell types (e.g., T cells).	10× Genomics, DNBSEQ-G400RS (MGI Tech)	mouse	[171,196,199]
**Potential therapeutic target recognition:** The E2 subunit of mitochondrial pyruvate dehydrogenase complex (PDC-E2) is potentially considered for validating potential immunotherapeutic candidate strategies against cholangiocarcinoma.	10× Genomics, DNBSEQ-G400RS (MGI Tech)	mouse	[171,196,199]
**Uncovering novel cell types:** The scRNA-seq technique reveals novel cell types and states without biased results. It identifies novel cell subtypes that undergo immune rejection. Primary biliary cholangitis liver and underlying developed cholangiocarcinoma contain several clonotypes, often shared between two tissues.	10× Genomics	mouse, human	[113,200]
**Tracking T-cell polarization:** scRNA-seq detects genes associated with Th1 and Tc1 lymphocyte subsets in primary biliary cholangitis compared with primary sclerosing cholangitis livers. These T cells were detected within cholangiocarcinoma tumors and draining lymph nodes of mice with primary biliary cholangitis but not primary sclerosing cholangitis. Th1- and Tc1-polarized subsets play a key role in rejecting cholangiocarcinoma tumors.	10× Genomics	mouse	[199]

**Table 12:** Examples of important biomarkers of tumor transplantation complications.

References	Methods/Technologies	Tumor Types	Donor/Receptor	Gene Biomarkers
[199]	10× Genomics	cholangiocarcinoma	mouse	FoxP3, IFNγ, IL4, IL17a, Cd3g, Cd4, Cd8a, Id2, Tcf7, Eomes Il7r, Prdm1, Il2, Tbx21, Gata3, Il4, Rorc, Bcl6, Foxp3, Gzma, Gzmb, Gzmk, Ifng, Icos, Cd28, Cd27, Tnfrsf4, Tnfrsf9, Tnfrsf18, Cd40lg, Pdcd1, Ctla4, Lag3, Havcr2, Tigit, Btla, Lta, Adora2a, Klrg1, Cd38, Nt5e,
[171]	DNBSEQ-G400RS (MGI Tech)	cholangiocarcinoma	mouse	Alb, Apoa1, Ass1, Spp1, Sox9
[196]	10× Genomics	melanoma tumors	human	CD2, CD3D, CD3E, CD3G, CD19, CD79A, CD79B, BLK, CD163, CD14, CSF1R, PECAM1, VWF, CDH5, FAP, THY1, DCN, COL1A1, COL1A2, COL6A1, COL6A2, COL6A3
[198]	10× Genomics	Non-small cell lung carcinoma (NSCLC), neuroblastoma (NB), MBC, glioblastoma; high-grade glioma, CLL, ovarian, melanoma, sarcoma	human	KRT8, MRC1, TRAC, JCHAIN, TPSAB1, PTPRC, APOE, MAG, THY1, MITF, CA8, CFH, PAX3, CD99, KRT5, SFTPB, FOXJ1, MUC1, CGRP, SFTPC, AGER, FSP1 PECAM1, TH, MYCN, SOX2, STMN2, FDX1, PROM1, PDGFRA, UCHL1, LGALS3, HOPX, VIM
[197]	10× Genomics	Non-small-cell lung cancer (NSCLC), lung squamous carcinoma (LUSC), lung adenocarcinoma (LUAD)	human	TPSAB1, TPSB2, CPA3, HPGDS, CLU, AREG, MS4A2, RGS13, VWA5A, LAPTM4A, C1orf186, SLC18A2, LTC4S, KIT, HDC, MAOB, RGS1, RP11- 354E11.2, SAMSN1, RGS2, SLC26A2, PTGS1, NSMCE1

## Data Availability

Data sharing is not applicable to this article as no new data were created or analyzed in this study.

## References

[1] Jia Q., Chu H., Jin Z., Long H., Zhu B. (2022). High-throughput single-cell sequencing in cancer research. Signal Transduct. Target. Ther..

[2] Eum H.H., Jeong D., Kim N., Jo A., Na M., Kang H., Hong Y., Kong J.S., Jeong G.H., Yoo S.A. (2024). Single-cell RNA sequencing reveals myeloid and T cell co-stimulation mediated by IL-7 anti-cancer immunotherapy. Br. J. Cancer.

[3] Eze U.C., Bhaduri A., Haeussler M., Nowakowski T.J., Kriegstein A.R. (2021). Single-cell atlas of early human brain development highlights heterogeneity of human neuroepithelial cells and early radial glia. Nat. Neurosci..

[4] Dopp J., Ortega A., Davie K., Poovathingal S., Baz E.-S., Liu S. (2024). Single-cell transcriptomics reveals that glial cells integrate homeostatic and circadian processes to drive sleep–wake cycles. Nat. Neurosci..

[5] Wu H., Malone A.F., Donnelly E.L., Kirita Y., Uchimura K., Ramakrishnan S.M., Gaut J.P., Humphreys B.D. (2018). Single-Cell Transcriptomics of a Human Kidney Allograft Biopsy Specimen Defines a Diverse Inflammatory Response. J. Am. Soc. Nephrol..

[6] Papalexi E., Satija R. (2018). Single-cell RNA sequencing to explore immune cell heterogeneity. Nat. Rev. Immunol..

[7] Tang Y., Wang J., Zhang Y., Li J., Chen M., Gao Y., Dai M., Lin S., He X., Wu C. (2022). Single-Cell RNA Sequencing Identifies Intra-Graft Population Heterogeneity in Acute Heart Allograft Rejection in Mouse. Front. Immunol..

[8] Thareja G., Muthukumar T. (2024). Partners in Crime: Inferring Cell-to-cell Interactions in Kidney Allograft Rejection from Single-cell RNA Sequencing. Transplantation.

[9] Elmentaite R., Ross A.D.B., Roberts K., James K.R., Ortmann D., Gomes T., Nayak K., Tuck L., Pritchard S., Bayraktar O.A. (2020). Single-Cell Sequencing of Developing Human Gut Reveals Transcriptional Links to Childhood Crohn’s Disease. Dev. Cell.

[10] Bye C.R., Penna V., de Luzy I.R., Gantner C.W., Hunt C.P.J., Thompson L.H., Parish C.L. (2019). Transcriptional Profiling of Xenogeneic Transplants: Examining Human Pluripotent Stem Cell-Derived Grafts in the Rodent Brain. Stem Cell Rep..

[11] Abedini-Nassab R., Pouryosef Miandoab M., Sasmaz M. (2021). Microfluidic Synthesis, Control, and Sensing of Magnetic Nanoparticles: A Review. Micromachines.

[12] Mantri M., Scuderi G.J., Abedini-Nassab R., Wang M.F.Z., McKellar D., Shi H., Grodner B., Butcher J.T., De Vlaminck I. (2021). Spatiotemporal single-cell RNA sequencing of developing chicken hearts identifies interplay between cellular differentiation and morphogenesis. Nat. Commun..

[13] Buchwald J.E., Martins P.N. (2022). Designer organs: The future of personalized transplantation. Artif. Organs.

[14] Shi T., Roskin K., Baker B.M., Woodle E.S., Hildeman D. (2021). Advanced Genomics-Based Approaches for Defining Allograft Rejection with Single Cell Resolution. Front. Immunol..

[15] Raza S.S., Wagner A.P., Hussain Y.S., Khan M.A. (2018). Mechanisms underlying dental-derived stem cell-mediated neurorestoration in neurodegenerative disorders. Stem Cell Res. Ther..

[16] McCauley H.A., Guasch G. (2013). Serial orthotopic transplantation of epithelial tumors in single-cell suspension. Methods Mol. Biol..

[17] Kim H.K., Ha T.W., Lee M.R. (2021). Single-Cell Transcriptome Analysis as a Promising Tool to Study Pluripotent Stem Cell Reprogramming. Int. J. Mol. Sci..

[18] Herzenberg L.A., Parks D., Sahaf B., Perez O., Roederer M., Herzenberg L.A. (2002). The history and future of the fluorescence activated cell sorter and flow cytometry: A view from Stanford. Clin. Chem..

[19] Bonner W.A., Hulett H.R., Sweet R.G., Herzenberg L.A. (1972). Fluorescence activated cell sorting. Rev. Sci. Instrum..

[20] McKinnon K.M. (2018). Flow Cytometry: An Overview. Curr. Protoc. Immunol..

[21] Koike Y., Kodera S., Yokoyama Y., Hayakawa T. (2022). Real-time irradiation system using patterned light to actuate light-driven on-chip gel actuators. Robomech J..

[22] Adam G., Chidambaram S., Reddy S.S., Ramani K., Cappelleri D.J. (2021). Towards a Comprehensive and Robust Micromanipulation System with Force-Sensing and VR Capabilities. Micromachines.

[23] Kato Y., Matsumoto T., Kino-Oka M. (2019). Effect of liquid flow by pipetting during medium change on deformation of hiPSC aggregates. Regen. Ther..

[24] Di Carlo D., Wu L.Y., Lee L.P. (2006). Dynamic single cell culture array. Lab. Chip.

[25] Narayanamurthy V., Nagarajan S., Firus Khan A.Y., Samsuri F., Sridhar T.M. (2017). Microfluidic hydrodynamic trapping for single cell analysis: Mechanisms, methods and applications. Anal. Methods.

[26] Luan Q., Macaraniag C., Zhou J., Papautsky I. (2020). Microfluidic systems for hydrodynamic trapping of cells and clusters. Biomicrofluidics.

[27] Abedini-Nassab R. (2020). Magnetophoretic Circuit Biocompatibility. J. Mech. Med. Biol..

[28] Ahmadi F., Tran H., Letourneau N., Little S.R., Fortin A., Moraitis A.N., Shih S.C.C. (2024). An Automated Single-Cell Droplet-Digital Microfluidic Platform for Monoclonal Antibody Discovery. Small.

[29] Abedini-Nassab R., Mahdaviyan N. (2020). A Microfluidic Platform Equipped with Magnetic Nano Films for Organizing Bio-Particle Arrays and Long-Term Studies. IEEE Sens. J..

[30] Abedini-Nassab R., Shourabi R. (2022). High-throughput precise particle transport at single-particle resolution in a three-dimensional magnetic field for highly sensitive bio-detection. Sci. Rep..

[31] Zhang Y., Zhou A., Chen S., Lum G.Z., Zhang X. (2022). A perspective on magnetic microfluidics: Towards an intelligent future. Biomicrofluidics.

[32] Xu H., Liao C., Zuo P., Liu Z., Ye B.C. (2018). Magnetic-Based Microfluidic Device for On-Chip Isolation and Detection of Tumor-Derived Exosomes. Anal. Chem..

[33] Abedini-Nassab R., Ding X., Xie H. (2022). A novel magnetophoretic-based device for magnetometry and separation of single magnetic particles and magnetized cells. Lab. Chip.

[34] Sadeghidelouei N., Abedini-Nassab R. (2023). Unidirectional particle transport in microfluidic chips operating in a tri-axial magnetic field for particle concentration and bio-analyte detection. Microfluid. Nanofluidics.

[35] Yu E.S., Lee H., Lee S.M., Kim J., Kim T., Lee J., Kim C., Seo M., Kim J.H., Byun Y.T. (2020). Precise capture and dynamic relocation of nanoparticulate biomolecules through dielectrophoretic enhancement by vertical nanogap architectures. Nat. Commun..

[36] Punjiya M., Nejad H.R., Mathews J., Levin M., Sonkusale S. (2019). A flow through device for simultaneous dielectrophoretic cell trapping and AC electroporation. Sci. Rep..

[37] Mugele F., Baret J.-C. (2005). Electrowetting: From basics to applications. J. Physics Condens. Matter.

[38] Abedini-Nassab R., Wirfel J., Talebjedi B., Tasnim N., Hoorfar M. (2022). Quantifying the dielectrophoretic force on colloidal particles in microfluidic devices. Microfluid. Nanofluidics.

[39] Yang S., Rufo J., Zhong R., Rich J., Wang Z., Lee L.P., Huang T.J. (2023). Acoustic tweezers for high-throughput single-cell analysis. Nat. Protoc..

[40] Abedini-Nassab R., Emami S.M., Nowghabi A.N. (2022). Nanotechnology and Acoustics in Medicine and Biology. Recent Pat. Nanotechnol..

[41] Rufo J., Cai F., Friend J., Wiklund M., Huang T.J. (2022). Acoustofluidics for biomedical applications. Nat. Rev. Methods Primers.

[42] Zhang Y., Chen X. (2021). Particle separation in microfluidics using different modal ultrasonic standing waves. Ultrason. Sonochem..

[43] Ohiri K.A., Kelly S.T., Motschman J.D., Lin K.H., Wood K.C., Yellen B.B. (2018). An acoustofluidic trap and transfer approach for organizing a high density single cell array. Lab. Chip.

[44] Volpe G., Maragò O.M., Rubinsztein-Dunlop H., Pesce G., Stilgoe A.B., Volpe G., Tkachenko G., Truong V.G., Chormaic S.N., Kalantarifard F. (2023). Roadmap for optical tweezers. J. Phys. Photonics.

[45] Wang M.M., Tu E., Raymond D.E., Yang J.M., Zhang H., Hagen N., Dees B., Mercer E.M., Forster A.H., Kariv I. (2005). Microfluidic sorting of mammalian cells by optical force switching. Nat. Biotechnol..

[46] Yang Y., Mao Y., Shin K.S., Chui C.O., Chiou P.Y. (2016). Self-Locking Optoelectronic Tweezers for Single-Cell and Microparticle Manipulation across a Large Area in High Conductivity Media. Sci. Rep..

[47] Schraivogel D., Kuhn T.M., Rauscher B., Rodriguez-Martinez M., Paulsen M., Owsley K., Middlebrook A., Tischer C., Ramasz B., Ordonez-Rueda D. (2022). High-speed fluorescence image-enabled cell sorting. Science.

[48] Valle M., O’Brien B., Green T.D., Reiner J.E., Seashols-Williams S. (2024). Droplet-based optical trapping for cell separation in mock forensic samples. J. Forensic Sci..

[49] Schneckenburger H., Hendinger A., Sailer R., Gschwend M.H., Strauss W.S., Bauer M., Schütze K. (2000). Cell viability in optical tweezers: High power red laser diode versus Nd:YAG laser. J. Biomed. Opt..

[50] Konishi S., Ohya C., Yamada T. (2021). Selective control of the contact and transport between droplet pairs by electrowetting-on-dielectric for droplet-array sandwiching technology. Sci. Rep..

[51] Vallet M., Berge B., Vovelle L. (1996). Electrowetting of water and aqueous solutions on poly(ethylene terephthalate) insulating films. Polymer.

[52] Abedini-Nassab R., Sadeghidelouei N., Shields Iv C.W. (2023). Magnetophoretic circuits: A review of device designs and implementation for precise single-cell manipulation. Anal. Chim. Acta.

[53] Abedini-Nassab R. (2023). Magnetomicrofluidic Circuits for Single-Bioparticle Transport.

[54] Abedini-Nassab R. (2019). Magnetomicrofluidic Platforms for Organizing Arrays of Single-Particles and Particle-Pairs. J. Microelectromech. Syst..

[55] Dashti R., Abedini-Nassab R. (2024). A High-Throughput Hybrid Electromicrofluidic Platform for Organizing Single-Cell Protein Secretion Profiling Assays. IEEE Sens. J..

[56] Au A.K., Lai H., Utela B.R., Folch A. (2011). Microvalves and Micropumps for BioMEMS. Micromachines.

[57] Studer V., Hang G., Pandolfi A., Ortiz M., French Anderson W., Quake S.R. (2003). Scaling properties of a low-actuation pressure microfluidic valve. J. Appl. Phys..

[58] Thorsen T., Maerkl S.J., Quake S.R. (2002). Microfluidic large-scale integration. Science.

[59] Marcus J.S., Anderson W.F., Quake S.R. (2006). Microfluidic single-cell mRNA isolation and analysis. Anal. Chem..

[60] Macosko E.Z., Basu A., Satija R., Nemesh J., Shekhar K., Goldman M., Tirosh I., Bialas A.R., Kamitaki N., Martersteck E.M. (2015). Highly Parallel Genome-wide Expression Profiling of Individual Cells Using Nanoliter Droplets. Cell.

[61] Jain A., Stavrakis S., deMello A. (2024). Droplet-based microfluidics and enzyme evolution. Curr. Opin. Biotechnol..

[62] Nan L., Zhang H., Weitz D.A., Shum H.C. (2024). Development and future of droplet microfluidics. Lab. Chip.

[63] Shi J., Zhang Y., Fan Y., Liu Y., Yang M. (2024). Recent advances in droplet-based microfluidics in liquid biopsy for cancer diagnosis. Droplet.

[64] Zhang J., Xu W., Xu F., Lu W., Hu L., Zhou J., Zhang C., Jiang Z. (2021). Microfluidic droplet formation in co-flow devices fabricated by micro 3D printing. J. Food Eng..

[65] Cramer C., Fischer P., Windhab E.J. (2004). Drop formation in a co-flowing ambient fluid. Chem. Eng. Sci..

[66] Yao J., Lin F., Kim H.S., Park J. (2019). The Effect of Oil Viscosity on Droplet Generation Rate and Droplet Size in a T-Junction Microfluidic Droplet Generator. Micromachines.

[67] Ushikubo F.Y., Birribilli F.S., Oliveira D.R.B., Cunha R.L. (2014). Y- and T-junction microfluidic devices: Effect of fluids and interface properties and operating conditions. Microfluid. Nanofluidics.

[68] Garstecki P., Fuerstman M.J., Stone H.A., Whitesides G.M. (2006). Formation of droplets and bubbles in a microfluidic T-junction—Scaling and mechanism of break-up. Lab. Chip.

[69] Yin Z., Huang Z., Lin X., Gao X., Bao F. (2020). Droplet Generation in a Flow-Focusing Microfluidic Device with External Mechanical Vibration. Micromachines.

[70] Dewandre A., Rivero-Rodriguez J., Vitry Y., Sobac B., Scheid B. (2020). Microfluidic droplet generation based on non-embedded co-flow-focusing using 3D printed nozzle. Sci. Rep..

[71] Bageritz J., Raddi G. (2019). Single-Cell RNA Sequencing with Drop-Seq. Methods Mol. Biol..

[72] Klein A.M., Mazutis L., Akartuna I., Tallapragada N., Veres A., Li V., Peshkin L., Weitz D.A., Kirschner M.W. (2015). Droplet barcoding for single-cell transcriptomics applied to embryonic stem cells. Cell.

[73] Lu Y., Shiau F., Yi W., Lu S., Wu Q., Pearson J.D., Kallman A., Zhong S., Hoang T., Zuo Z. (2020). Single-Cell Analysis of Human Retina Identifies Evolutionarily Conserved and Species-Specific Mechanisms Controlling Development. Dev. Cell.

[74] Gierahn T.M., Wadsworth M.H., Hughes T.K., Bryson B.D., Butler A., Satija R., Fortune S., Love J.C., Shalek A.K. (2017). Seq-Well: Portable, low-cost RNA sequencing of single cells at high throughput. Nat. Methods.

[75] Ramskold D., Luo S., Wang Y.C., Li R., Deng Q., Faridani O.R., Daniels G.A., Khrebtukova I., Loring J.F., Laurent L.C. (2020). Author Correction: Full-length mRNA-Seq from single-cell levels of RNA and individual circulating tumor cells. Nat. Biotechnol..

[76] Isakova A., Neff N., Quake S.R. (2021). Single-cell quantification of a broad RNA spectrum reveals unique noncoding patterns associated with cell types and states. Proc. Natl. Acad. Sci. USA.

[77] Picelli S., Faridani O.R., Björklund Å.K., Winberg G., Sagasser S., Sandberg R. (2014). Full-length RNA-seq from single cells using Smart-seq2. Nat. Protoc..

[78] Picelli S., Björklund Å.K., Faridani O.R., Sagasser S., Winberg G., Sandberg R. (2013). Smart-seq2 for sensitive full-length transcriptome profiling in single cells. Nat. Methods.

[79] Hagemann-Jensen M., Ziegenhain C., Chen P., Ramsköld D., Hendriks G.-J., Larsson A.J.M., Faridani O.R., Sandberg R. (2020). Single-cell RNA counting at allele and isoform resolution using Smart-seq3. Nat. Biotechnol..

[80] Cao J., Packer J.S., Ramani V., Cusanovich D.A., Huynh C., Daza R., Qiu X., Lee C., Furlan S.N., Steemers F.J. (2017). Comprehensive single-cell transcriptional profiling of a multicellular organism. Science.

[81] Sheng K., Cao W., Niu Y., Deng Q., Zong C. (2017). Effective detection of variation in single-cell transcriptomes using MATQ-seq. Nat. Methods.

[82] Hashimshony T., Wagner F., Sher N., Yanai I. (2012). CEL-Seq: Single-cell RNA-Seq by multiplexed linear amplification. Cell Rep..

[83] Hashimshony T., Senderovich N., Avital G., Klochendler A., de Leeuw Y., Anavy L., Gennert D., Li S., Livak K.J., Rozenblatt-Rosen O. (2016). CEL-Seq2: Sensitive highly-multiplexed single-cell RNA-Seq. Genome Biol..

[84] Branton D., Deamer D.W., Marziali A., Bayley H., Benner S.A., Butler T., Di Ventra M., Garaj S., Hibbs A., Huang X. (2008). The potential and challenges of nanopore sequencing. Nat. Biotechnol..

[85] Philpott M., Watson J., Thakurta A., Brown T., Brown T., Oppermann U., Cribbs A.P. (2021). Nanopore sequencing of single-cell transcriptomes with scCOLOR-seq. Nat. Biotechnol..

[86] Shiau C.K., Lu L., Kieser R., Fukumura K., Pan T., Lin H.Y., Yang J., Tong E.L., Lee G., Yan Y. (2023). High throughput single cell long-read sequencing analyses of same-cell genotypes and phenotypes in human tumors. Nat. Commun..

[87] Abedini-Nassab R. (2017). Nanotechnology and Nanopore Sequencing. Recent. Pat. Nanotechnol..

[88] Wang Y., Zhao Y., Bollas A., Wang Y., Au K.F. (2021). Nanopore sequencing technology, bioinformatics and applications. Nat. Biotechnol..

[89] Louie S.M., Moye A.L., Wong I.G., Lu E., Shehaj A., Garcia-de-Alba C., Ararat E., Raby B.A., Lu B., Paschini M. (2022). Progenitor potential of lung epithelial organoid cells in a transplantation model. Cell Rep..

[90] Kono N., Arakawa K. (2019). Nanopore sequencing: Review of potential applications in functional genomics. Dev. Growth Differ..

[91] Koenig A.L., Shchukina I., Amrute J., Andhey P.S., Zaitsev K., Lai L., Bajpai G., Bredemeyer A., Smith G., Jones C. (2022). Single-cell transcriptomics reveals cell-type-specific diversification in human heart failure. Nat. Cardiovasc. Res..

[92] Paik D.T., Tian L., Williams I.M., Rhee S., Zhang H., Liu C., Mishra R., Wu S.M., Red-Horse K., Wu J.C. (2020). Single-Cell RNA Sequencing Unveils Unique Transcriptomic Signatures of Organ-Specific Endothelial Cells. Circulation.

[93] Cai J., Deng J., Gu W., Ni Z., Liu Y., Kamra Y., Saxena A., Hu Y., Yuan H., Xiao Q. (2020). Impact of Local Alloimmunity and Recipient Cells in Transplant Arteriosclerosis. Circ. Res..

[94] Kopecky B.J., Dun H., Amrute J.M., Lin C.Y., Bredemeyer A.L., Terada Y., Bayguinov P.O., Koenig A.L., Frye C.C., Fitzpatrick J.A.J. (2022). Donor Macrophages Modulate Rejection after Heart Transplantation. Circulation.

[95] Hu Z., Liu W., Hua X., Chen X., Chang Y., Hu Y., Xu Z., Song J. (2021). Single-Cell Transcriptomic Atlas of Different Human Cardiac Arteries Identifies Cell Types Associated with Vascular Physiology. Arterioscler. Thromb. Vasc. Biol..

[96] Anto Michel N., Ljubojevic-Holzer S., Bugger H., Zirlik A. (2022). Cellular Heterogeneity of the Heart. Front. Cardiovasc. Med..

[97] Wan J., Zhang Z., Tian S., Huang S., Jin H., Liu X., Zhang W. (2022). Single cell study of cellular diversity and mutual communication in chronic heart failure and drug repositioning. Genomics.

[98] Chen Z., Xu H., Li Y., Zhang X., Cui J., Zou Y., Yu J., Wu J., Xia J. (2022). Single-Cell RNA sequencing reveals immune cell dynamics and local intercellular communication in acute murine cardiac allograft rejection. Theranostics.

[99] Martini E., Kunderfranco P., Peano C., Carullo P., Cremonesi M., Schorn T., Carriero R., Termanini A., Colombo F.S., Jachetti E. (2019). Single-Cell Sequencing of Mouse Heart Immune Infiltrate in Pressure Overload–Driven Heart Failure Reveals Extent of Immune Activation. Circulation.

[100] Kong D., Huang S., Miao X., Li J., Wu Z., Shi Y., Liu H., Jiang Y., Yu X., Xie M. (2023). The dynamic cellular landscape of grafts with acute rejection after heart transplantation. J. Heart Lung Transplant..

[101] Jung Y., Kim J., Jang H., Kim G., Kwon Y.-W. (2023). Strategy of Patient-Specific Therapeutics in Cardiovascular Disease through Single-Cell RNA Sequencing. Korean Circ. J..

[102] Loupy A., Duong Van Huyen J.P., Hidalgo L., Reeve J., Racapé M., Aubert O., Venner J.M., Falmuski K., Bories M.C., Beuscart T. (2017). Gene Expression Profiling for the Identification and Classification of Antibody-Mediated Heart Rejection. Circulation.

[103] Hua X., Hu G., Hu Q., Chang Y., Hu Y., Gao L., Chen X., Yang P.-C., Zhang Y., Li M. (2020). Single-Cell RNA Sequencing to Dissect the Immunological Network of Autoimmune Myocarditis. Circulation.

[104] Yang L., Zhu Y., Tian D., Wang S., Guo J., Sun G., Jin H., Zhang C., Shi W., Gershwin M.E. (2021). Transcriptome landscape of double negative T cells by single-cell RNA sequencing. J. Autoimmun..

[105] Schumacher D., Kramann R. (2023). Multiomic Spatial Mapping of Myocardial Infarction and Implications for Personalized Therapy. Arterioscler. Thromb. Vasc. Biol..

[106] Clark A.R., Greka A. (2020). The power of one: Advances in single-cell genomics in the kidney. Nat. Rev. Nephrol..

[107] Wilson P.C., Wu H., Kirita Y., Uchimura K., Ledru N., Rennke H.G., Welling P.A., Waikar S.S., Humphreys B.D. (2019). The single-cell transcriptomic landscape of early human diabetic nephropathy. Proc. Natl. Acad. Sci. USA.

[108] Young M.D., Mitchell T.J., Vieira Braga F.A., Tran M.G.B., Stewart B.J., Ferdinand J.R., Collord G., Botting R.A., Popescu D.M., Loudon K.W. (2018). Single-cell transcriptomes from human kidneys reveal the cellular identity of renal tumors. Science.

[109] Muto Y., Wilson P.C., Ledru N., Wu H., Dimke H., Waikar S.S., Humphreys B.D. (2021). Single cell transcriptional and chromatin accessibility profiling redefine cellular heterogeneity in the adult human kidney. Nat. Commun..

[110] Shen Q., Wang Y., Chen J., Ma L., Huang X., Tang S.C.W., Lan H., Jiang H. (2021). Single-Cell RNA Sequencing Reveals the Immunological Profiles of Renal Allograft Rejection in Mice. Front. Immunol..

[111] Shi T., Burg A.R., Caldwell J.T., Roskin K.M., Castro-Rojas C.M., Chukwuma P.C., Gray G.I., Foote S.G., Alonso J.A., Cuda C.M. (2023). Single-cell transcriptomic analysis of renal allograft rejection reveals insights into intragraft TCR clonality. J. Clin. Investig..

[112] Malone A.F., Wu H., Fronick C., Fulton R., Gaut J.P., Humphreys B.D. (2020). Harnessing Expressed Single Nucleotide Variation and Single Cell RNA Sequencing To Define Immune Cell Chimerism in the Rejecting Kidney Transplant. J. Am. Soc. Nephrol..

[113] Liu Y., Hu J., Liu D., Zhou S., Liao J., Liao G., Yang S., Guo Z., Li Y., Li S. (2020). Single-cell analysis reveals immune landscape in kidneys of patients with chronic transplant rejection. Theranostics.

[114] Zhuang Q., Li H., Peng B., Liu Y., Zhang Y., Cai H., Liu S., Ming Y. (2021). Single-Cell Transcriptomic Analysis of Peripheral Blood Reveals a Novel B-Cell Subset in Renal Allograft Recipients with Accommodation. Front. Pharmacol..

[115] Asano Y., Daccache J., Jain D., Ko K., Kinloch A., Veselits M., Wolfgeher D., Chang A., Josephson M., Cunningham P. (2021). Innate-like self-reactive B cells infiltrate human renal allografts during transplant rejection. Nat. Commun..

[116] Lamarthee B., Callemeyn J., Van Herck Y., Antoranz A., Anglicheau D., Boada P., Becker J.U., Debyser T., De Smet F., De Vusser K. (2023). Transcriptional and spatial profiling of the kidney allograft unravels a central role for FcyRIII+ innate immune cells in rejection. Nat. Commun..

[117] van der List A.C.J., Litjens N.H.R., Brouwer R.W.W., Klepper M., den Dekker A.T., van Ijcken W.F.J., Betjes M.G.H. (2023). Single-Cell RNA Sequencing of Donor-Reactive T Cells Reveals Role of Apoptosis in Donor-Specific Hyporesponsiveness of Kidney Transplant Recipients. Int. J. Mol. Sci..

[118] Dangi A., Natesh N.R., Husain I., Ji Z., Barisoni L., Kwun J., Shen X., Thorp E.B., Luo X. (2020). Single cell transcriptomics of mouse kidney transplants reveals a myeloid cell pathway for transplant rejection. JCI Insight.

[119] Wang J., Luo P., Zhao J., Tan J., Huang F., Ma R., Huang P., Huang M., Huang Y., Wei Q. (2020). Profiling the Resident and Infiltrating Monocyte/Macrophages during Rejection following Kidney Transplantation. J. Immunol. Res..

[120] Subramanian A., Sidhom E.H., Emani M., Vernon K., Sahakian N., Zhou Y., Kost-Alimova M., Slyper M., Waldman J., Dionne D. (2019). Single cell census of human kidney organoids shows reproducibility and diminished off-target cells after transplantation. Nat. Commun..

[121] Garreta E., Nauryzgaliyeva Z., Montserrat N. (2021). Human induced pluripotent stem cell-derived kidney organoids toward clinical implementations. Curr. Opin. Biomed. Eng..

[122] Melo Ferreira R., Sabo A.R., Winfree S., Collins K.S., Janosevic D., Gulbronson C.J., Cheng Y.H., Casbon L., Barwinska D., Ferkowicz M.J. (2021). Integration of spatial and single-cell transcriptomics localizes epithelial cell-immune cross-talk in kidney injury. JCI Insight.

[123] Zheng Y., Lu P., Deng Y., Wen L., Wang Y., Ma X., Wang Z., Wu L., Hong Q., Duan S. (2020). Single-Cell Transcriptomics Reveal Immune Mechanisms of the Onset and Progression of IgA Nephropathy. Cell Rep..

[124] Lubetzky M.L., Salinas T., Schwartz J.E., Suthanthiran M. (2021). Urinary Cell mRNA Profiles Predictive of Human Kidney Allograft Status. Clin. J. Am. Soc. Nephrol..

[125] Azim S., Zubair H., Rousselle T., McDaniels J.M., Shetty A.C., Kuscu C., Kuscu C., Talwar M., Eason J.D., Maluf D.G. (2023). Single-cell RNA sequencing reveals peripheral blood mononuclear immune cell landscape associated with operational tolerance in a kidney transplant recipient. Am. J. Transplant..

[126] Muthukumar T., Yang H., Belkadi A., Thareja G., Li C., Snopkowski C., Chen K., Salinas T., Lubetzky M., Lee J. (2021). Single Cell Rna-Sequencing of Urinary Cells and Defining the Immune Landscape of Rejection in Human Kidney Allografts. Am. J. Transplant.

[127] Kong F., Ye S., Zhong Z., Zhou X., Zhou W., Liu Z., Lan J., Xiong Y., Ye Q. (2021). Single-Cell Transcriptome Analysis of Chronic Antibody-Mediated Rejection after Renal Transplantation. Front. Immunol..

[128] Wen N., Wu J., Li H., Liao J., Lan L., Yang X., Zhu G., Lei Z., Dong J., Sun X. (2023). Immune landscape in rejection of renal transplantation revealed by high-throughput single-cell RNA sequencing. Front. Cell Dev. Biol..

[129] Suryawanshi H., Yang H., Lubetzky M., Morozov P., Lagman M., Thareja G., Alonso A., Li C., Snopkowski C., Belkadi A. (2022). Detection of infiltrating fibroblasts by single-cell transcriptomics in human kidney allografts. PLoS ONE.

[130] Pang Q., Chen L., An C., Zhou J., Xiao H. (2024). Single-cell and bulk RNA sequencing highlights the role of M1-like infiltrating macrophages in antibody-mediated rejection after kidney transplantation. Heliyon.

[131] Park J., Shrestha R., Qiu C., Kondo A., Huang S., Werth M., Li M., Barasch J., Suszták K. (2018). Single-cell transcriptomics of the mouse kidney reveals potential cellular targets of kidney disease. Science.

[132] Dell’Orso S., Juan A.H., Ko K.D., Naz F., Perovanovic J., Gutierrez-Cruz G., Feng X., Sartorelli V. (2019). Correction: Single cell analysis of adult mouse skeletal muscle stem cells in homeostatic and regenerative conditions. Development.

[133] Pellin D., Loperfido M., Baricordi C., Wolock S.L., Montepeloso A., Weinberg O.K., Biffi A., Klein A.M., Biasco L. (2019). A comprehensive single cell transcriptional landscape of human hematopoietic progenitors. Nat. Commun..

[134] Rashmi P., Sur S., Sigdel T.K., Boada P., Schroeder A.W., Damm I., Kretzler M., Hodgin J., Hartoularos G., Ye C.J. (2022). Multiplexed droplet single-cell sequencing (Mux-Seq) of normal and transplant kidney. Am. J. Transplant..

[135] Wilson G.W., Moshkelgosha S., Duong A., Keshavjee S., Martinu T., Juvet S., Yeung J.C. (2021). Deconvolution of Donor and Recipient Cells from Lung Transplant Single Cell RNA-seq Data. J. Heart Lung Transplant..

[136] Smirnova N.F., Riemondy K., Bueno M., Collins S., Suresh P., Wang X., Patel K.N., Cool C., Königshoff M., Sharma N.S. (2022). Single-cell transcriptome mapping identifies a local, innate B cell population driving chronic rejection after lung transplantation. JCI Insight.

[137] Snyder M.E., Finlayson M.O., Connors T.J., Dogra P., Senda T., Bush E., Carpenter D., Marboe C., Benvenuto L., Shah L. (2019). Generation and persistence of human tissue-resident memory T cells in lung transplantation. Sci. Immunol..

[138] Bharat A., Querrey M., Markov N.S., Kim S., Kurihara C., Garza-Castillon R., Manerikar A., Shilatifard A., Tomic R., Politanska Y. (2020). Lung transplantation for patients with severe COVID-19. Sci. Transl. Med..

[139] Wanczyk H., Jensen T., Weiss D.J., Finck C. (2021). Advanced single-cell technologies to guide the development of bioengineered lungs. Am. J. Physiol. Lung Cell. Mol. Physiol..

[140] Mahata B., Zhang X., Kolodziejczyk A.A., Proserpio V., Haim-Vilmovsky L., Taylor A.E., Hebenstreit D., Dingler F.A., Moignard V., Göttgens B. (2014). Single-cell RNA sequencing reveals T helper cells synthesizing steroids de novo to contribute to immune homeostasis. Cell Rep..

[141] Hurskainen M., Mižíková I., Cook D.P., Andersson N., Cyr-Depauw C., Lesage F., Helle E., Renesme L., Jankov R.P., Heikinheimo M. (2021). Single cell transcriptomic analysis of murine lung development on hyperoxia-induced damage. Nat. Commun..

[142] Travaglini K.J., Nabhan A.N., Penland L., Sinha R., Gillich A., Sit R.V., Chang S., Conley S.D., Mori Y., Seita J. (2020). A molecular cell atlas of the human lung from single-cell RNA sequencing. Nature.

[143] Silva T.d., Voisey J., Hopkins P., Apte S., Chambers D., O’Sullivan B. (2022). Markers of rejection of a lung allograft: State of the art. Biomark. Med..

[144] Misharin A.V., Morales-Nebreda L., Reyfman P.A., Cuda C.M., Walter J.M., McQuattie-Pimentel A.C., Chen C.-I., Anekalla K.R., Joshi N., Williams K.J. (2017). Monocyte-derived alveolar macrophages drive lung fibrosis and persist in the lung over the life span. J. Exp. Med..

[145] Lunardi F., Abbrescia D.I., Vedovelli L., Pezzuto F., Fortarezza F., Comacchio G.M., Guzzardo V., Ferrigno P., Loy M., Giraudo C. (2023). Molecular Profiling of Tissue Samples with Chronic Rejection from Patients with Chronic Lung Allograft Dysfunction: A Pilot Study in Cystic Fibrosis Patients. Biomolecules.

[146] Malone A.F. (2021). Monocytes and Macrophages in Kidney Transplantation and Insights from Single Cell RNA-Seq Studies. Kidney360.

[147] Lavin Y., Winter D., Blecher-Gonen R., David E., Keren-Shaul H., Merad M., Jung S., Amit I. (2014). Tissue-resident macrophage enhancer landscapes are shaped by the local microenvironment. Cell.

[148] Reyfman P.A., Walter J.M., Joshi N., Anekalla K.R., McQuattie-Pimentel A.C., Chiu S., Fernandez R., Akbarpour M., Chen C.-I., Ren Z. (2018). Single-Cell Transcriptomic Analysis of Human Lung Provides Insights into the Pathobiology of Pulmonary Fibrosis. Am. J. Respir. Crit. Care Med..

[149] Snyder M.E., Moghbeli K., Bondonese A., Craig A., Popescu I., Fan L., Tabib T., Lafyatis R., Chen K., Trejo Bittar H.E. (2021). Human lung tissue resident memory T cells are re-programmed but not eradicated with systemic glucocorticoids after acute cellular rejection. medRxiv.

[150] Lee S.M.L., Bertinetti-Lapatki C., Schiergens T.S., Jauch K.W., Roth A.B., Thasler W.E. (2020). Concurrent isolation of hepatic stem cells and hepatocytes from the human liver. In Vitro Cell Dev. Biol. Anim..

[151] Shi W., Wang Y., Zhang C., Jin H., Zeng Z., Wei L., Tian Y., Zhang D., Sun G. (2020). Isolation and purification of immune cells from the liver. Int. Immunopharmacol..

[152] MacParland S.A., Liu J.C., Ma X.Z., Innes B.T., Bartczak A.M., Gage B.K., Manuel J., Khuu N., Echeverri J., Linares I. (2018). Single cell RNA sequencing of human liver reveals distinct intrahepatic macrophage populations. Nat. Commun..

[153] Wang L., Li J., He S., Liu Y., Chen H., Yin M., Zou D., Chen S., Luo T., Yu X. (2021). Resolving the graft ischemia-reperfusion injury during liver transplantation at the single cell resolution. Cell Death Dis..

[154] Yang X., Lu D., Wang R., Lian Z., Lin Z., Zhuo J., Chen H., Yang M., Tan W., Wei X. (2021). Single-cell profiling reveals distinct immune phenotypes that contribute to ischaemia-reperfusion injury after steatotic liver transplantation. Cell Prolif..

[155] Wang Z., Shao X., Wang K., Lu X., Zhuang L., Yang X., Zhang P., Yang P., Zheng S., Xu X. (2022). Single-cell analysis reveals a pathogenic cellular module associated with early allograft dysfunction after liver transplantation. bioRxiv.

[156] Huang H., Chen R., Lin Y., Jiang J., Feng S., Zhang X., Zhang C., Ji Q., Chen H., Xie H. (2023). Decoding Single-cell Landscape and Intercellular Crosstalk in the Transplanted Liver. Transplantation.

[157] Morrison J.K., DeRossi C., Alter I.L., Nayar S., Giri M., Zhang C., Cho J.H., Chu J. (2022). Single-cell transcriptomics reveals conserved cell identities and fibrogenic phenotypes in zebrafish and human liver. Hepatol. Commun..

[158] Li X., Li S., Wu B., Xu Q., Teng D., Yang T., Sun Y., Zhao Y., Li T., Liu D. (2022). Landscape of immune cells heterogeneity in liver transplantation by single-cell RNA sequencing analysis. Front. Immunol..

[159] Shan Y., Qi D., Zhang L., Wu L., Li W., Liu H., Li T., Fu Z., Bao H., Song S. (2023). Single-cell RNA-seq revealing the immune features of donor liver during liver transplantation. Front. Immunol..

[160] Tang H., Yuan J., Gong Y.-F., Zhang C.-Y., Liu M., Luo S.-X. (2022). Single-cell transcriptome sequencing reveals potential novel combination of biomarkers for antibody-based cancer therapeutics in hepatocellular carcinoma. Front. Genet..

[161] Hautz T., Salcher S., Fodor M., Sturm G., Ebner S., Mair A., Trebo M., Untergasser G., Sopper S., Cardini B. (2023). Immune cell dynamics deconvoluted by single-cell RNA sequencing in normothermic machine perfusion of the liver. Nat. Commun..

[162] Zhao J., Zhang S., Liu Y., He X., Qu M., Xu G., Wang H., Huang M., Pan J., Liu Z. (2020). Single-cell RNA sequencing reveals the heterogeneity of liver-resident immune cells in human. Cell Discov..

[163] Ramachandran P., Dobie R., Wilson-Kanamori J., Dora E., Henderson B., Luu N., Portman J., Matchett K., Brice M., Marwick J. (2019). Resolving the fibrotic niche of human liver cirrhosis at single-cell level. Nature.

[164] Aizarani N., Saviano A., Mailly L., Durand S., Herman J.S., Pessaux P., Baumert T.F., Grün D. (2019). A human liver cell atlas reveals heterogeneity and epithelial progenitors. Nature.

[165] Zhang Q., He Y., Luo N., Patel S.J., Han Y., Gao R., Modak M., Carotta S., Haslinger C., Kind D. (2019). Landscape and dynamics of single immune cells in hepatocellular carcinoma. Cell.

[166] Barbetta A., Rocque B., Sarode D., Bartlett J.A., Emamaullee J. (2023). Revisiting transplant immunology through the lens of single-cell technologies. Semin. Immunopathol..

[167] Roushansarai N.S., Pascher A., Becker F. (2022). Innate Immune Cells during Machine Perfusion of Liver Grafts—The Janus Face of Hepatic Macrophages. J. Clin. Med..

[168] Tamburini B.A.J., Finlon J.M., Gillen A.E., Kriss M.S., Riemondy K.A., Fu R., Schuyler R.P., Hesselberth J.R., Rosen H.R., Burchill M.A. (2019). Chronic liver disease in humans causes expansion and differentiation of liver lymphatic endothelial cells. Front. Immunol..

[169] Dobie R., Wilson-Kanamori J.R., Henderson B.E., Smith J.R., Matchett K.P., Portman J.R., Wallenborg K., Picelli S., Zagorska A., Pendem S.V. (2019). Single-cell transcriptomics uncovers zonation of function in the mesenchyme during liver fibrosis. Cell Rep..

[170] Loeuillard E., Yang J., Buckarma E., Wang J., Liu Y., Conboy C., Pavelko K.D., Li Y., O’Brien D., Wang C. (2020). Targeting tumor-associated macrophages and granulocytic myeloid-derived suppressor cells augments PD-1 blockade in cholangiocarcinoma. J. Clin. Investig..

[171] Shiode Y., Kodama T., Shigeno S., Murai K., Tanaka S., Newberg J.Y., Kondo J., Kobayashi S., Yamada R., Hikita H. (2022). TNF receptor–related factor 3 inactivation promotes the development of intrahepatic cholangiocarcinoma through NF-κB-inducing kinase–mediated hepatocyte transdifferentiation. Hepatology.

[172] Frazzette N., Khodadadi-Jamayran A., Doudican N., Santana A., Felsen D., Pavlick A.C., Tsirigos A., Carucci J.A. (2020). Decreased cytotoxic T cells and TCR clonality in organ transplant recipients with squamous cell carcinoma. NPJ Precis. Oncol..

[173] Blau H.M., Daley G.Q. (2019). Stem Cells in the Treatment of Disease. N. Engl. J. Med..

[174] Karam D., Gertz M., Lacy M., Dispenzieri A., Hayman S., Dingli D., Buadi F., Kapoor P., Kourelis T., Warsame R. (2022). Impact of maintenance therapy post autologous stem cell transplantation for multiple myeloma in early and delayed transplant. Bone Marrow Transplant..

[175] Fast E.M., Sporrij A., Manning M., Rocha E.L., Yang S., Zhou Y., Guo J., Baryawno N., Barkas N., Scadden D. (2021). External signals regulate continuous transcriptional states in hematopoietic stem cells. Elife.

[176] Rahman M., Wang Z.Y., Li J.X., Xu H.W., Wang R., Wu Q. (2022). Single-Cell RNA Sequencing Reveals the Interaction of Injected ADSCs with Lung-Originated Cells in Mouse Pulmonary Fibrosis. Stem Cells Int..

[177] Zhao Y., Li X., Zhao W., Wang J., Yu J., Wan Z., Gao K., Yi G., Wang X., Fan B. (2019). Single-cell transcriptomic landscape of nucleated cells in umbilical cord blood. Gigascience.

[178] Wang F., Tan P., Zhang P., Ren Y., Zhou J., Li Y., Hou S., Li S., Zhang L., Ma Y. (2022). Single-cell architecture and functional requirement of alternative splicing during hematopoietic stem cell formation. Sci. Adv..

[179] Wittenbecher F., Keilholz L., Obermayer B., Conrad T., Frentsch M., Blau I.W., Vuong L.G., Borchert F., Lesch S., Movasshagi K. (2021). Single-Cell Clonal Tracking in Allogeneic Hematopoietic Stem Cell Transplantation Reveals Time Dependent and Distinct Functional Patterns in Traceable Donor T Cell Clones. Blood.

[180] Augsornworawat P., Maxwell K.G., Velazco-Cruz L., Millman J.R. (2020). Single-cell transcriptome profiling reveals β cell maturation in stem cell-derived islets after transplantation. Cell Rep..

[181] Tiklová K., Nolbrant S., Fiorenzano A., Björklund Å., Sharma Y., Heuer A., Gillberg L., Hoban D.B., Cardoso T., Adler A.F. (2020). Single cell transcriptomics identifies stem cell-derived graft composition in a model of Parkinson’s disease. Nat. Commun..

[182] Arjona M., Goshayeshi A., Rodriguez-Mateo C., Brett J.O., Both P., Ishak H., Rando T.A. (2022). Tubastatin A maintains adult skeletal muscle stem cells in a quiescent state ex vivo and improves their engraftment ability in vivo. Stem Cell Rep..

[183] Montarras D., Morgan J., Collins C., Relaix F., Zaffran S., Cumano A., Partridge T., Buckingham M. (2005). Direct isolation of satellite cells for skeletal muscle regeneration. Science.

[184] Dong F., Hao S., Zhang S., Zhu C., Cheng H., Yang Z., Hamey F.K., Wang X., Gao A., Wang F. (2020). Differentiation of transplanted haematopoietic stem cells tracked by single-cell transcriptomic analysis. Nat. Cell Biol..

[185] de Almeida G.P., Lichtner P., Eckstein G., Brinkschmidt T., Chu C.-F., Sun S., Reinhard J., Mädler S.C., Kloeppel M., Verbeek M. (2022). Human skin-resident host T cells can persist long term after allogeneic stem cell transplantation and maintain recirculation potential. Sci. Immunol..

[186] Sun C., Wang H., Ma Q., Chen C., Yue J., Li B., Zhang X. (2021). Time-course single-cell RNA sequencing reveals transcriptional dynamics and heterogeneity of limbal stem cells derived from human pluripotent stem cells. Cell Biosci..

[187] Grover A., Sanjuan-Pla A., Thongjuea S., Carrelha J., Giustacchini A., Gambardella A., Macaulay I., Mancini E., Luis T.C., Mead A. (2016). Single-cell RNA sequencing reveals molecular and functional platelet bias of aged haematopoietic stem cells. Nat. Commun..

[188] Zheng G.X., Terry J.M., Belgrader P., Ryvkin P., Bent Z.W., Wilson R., Ziraldo S.B., Wheeler T.D., McDermott G.P., Zhu J. (2017). Massively parallel digital transcriptional profiling of single cells. Nat. Commun..

[189] Maxwell K.G., Augsornworawat P., Velazco-Cruz L., Kim M.H., Asada R., Hogrebe N.J., Morikawa S., Urano F., Millman J.R. (2020). Gene-edited human stem cell–derived β cells from a patient with monogenic diabetes reverse preexisting diabetes in mice. Sci. Transl. Med..

[190] Oguma Y., Kuroda Y., Wakao S., Kushida Y., Dezawa M. (2022). Single-cell RNA sequencing reveals different signatures of mesenchymal stromal cell pluripotent-like and multipotent populations. iScience.

[191] Cesaro A., Defrene J., Lachhab A., Page N., Tardif M.R., Al-Shami A., Oravecz T., Fortin P.R., Daudelin J.F., Labrecque N. (2019). Enhanced myelopoiesis and aggravated arthritis in *S100a8*-deficient mice. PLoS ONE.

[192] Bode D., Cull A.H., Rubio-Lara J.A., Kent D.G. (2021). Exploiting Single-Cell Tools in Gene and Cell Therapy. Front. Immunol..

[193] You G., Zhang M., Bian Z., Guo H., Xu Z., Ni Y., Lan Y., Yue W., Gong Y., Chang Y. (2022). Decoding lymphomyeloid divergence and immune hyporesponsiveness in G-CSF-primed human bone marrow by single-cell RNA-seq. Cell Discov..

[194] Wisdom A.J., Mowery Y.M., Hong C.S., Himes J.E., Nabet B.Y., Qin X., Zhang D., Chen L., Fradin H., Patel R. (2020). Single cell analysis reveals distinct immune landscapes in transplant and primary sarcomas that determine response or resistance to immunotherapy. Nat. Commun..

[195] Sinha V.C., Rinkenbaugh A.L., Xu M., Zhou X., Zhang X., Jeter-Jones S., Shao J., Qi Y., Zebala J.A., Maeda D.Y. (2021). Single-cell evaluation reveals shifts in the tumor-immune niches that shape and maintain aggressive lesions in the breast. Nat. Commun..

[196] Tirosh I., Izar B., Prakadan S.M., Wadsworth M.H., Treacy D., Trombetta J.J., Rotem A., Rodman C., Lian C., Murphy G. (2016). Dissecting the multicellular ecosystem of metastatic melanoma by single-cell RNA-seq. Science.

[197] Lambrechts D., Wauters E., Boeckx B., Aibar S., Nittner D., Burton O., Bassez A., Decaluwé H., Pircher A., Van den Eynde K. (2018). Phenotype molding of stromal cells in the lung tumor microenvironment. Nat. Med..

[198] Slyper M., Porter C.B., Ashenberg O., Waldman J., Drokhlyansky E., Wakiro I., Smillie C., Smith-Rosario G., Wu J., Dionne D. (2020). A single-cell and single-nucleus RNA-Seq toolbox for fresh and frozen human tumors. Nat. Med..

[199] Paillet J., Plantureux C., Lévesque S., Le Naour J., Stoll G., Sauvat A., Caudana P., Tosello Boari J., Bloy N., Lachkar S. (2021). Autoimmunity affecting the biliary tract fuels the immunosurveillance of cholangiocarcinoma. J. Exp. Med..

[200] Noé A., Cargill T.N., Nielsen C.M., Russell A.J.C., Barnes E. (2020). The Application of Single-Cell RNA Sequencing in Vaccinology. J. Immunol. Res..

